# Effects of a smartphone app-augmented treatment for children with oppositional defiant disorder / conduct disorder and peer-related aggressive behavior – a pilot study

**DOI:** 10.1186/s13063-022-06325-6

**Published:** 2022-07-08

**Authors:** Anja Görtz-Dorten, Marlin Frank, Anja Fessel, Leonie Hofmann, Manfred Döpfner

**Affiliations:** 1 Department of Child and Adolescent Psychiatry, Psychosomatics and Psychotherapy, Faculty of Medicine and University Hospital Cologne, University of Cologne, Cologne, Germany; 2 School of Child and Adolescent Cognitive Behavior Therapy (AKiP), Faculty of Medicine and University Hospital Cologne, University of Cologne, Cologne, Germany

**Keywords:** Smartphone app, Children, Aggressive behavior, Cognitive behavioral therapy, Therapy homework, Adherence

## Abstract

**Background:**

Social competence training interventions, especially child-focused ones, have proven to be effective in the treatment of children with conduct disorder. Therapy homework assignments implemented between the therapy sessions are essential for practicing strategies developed during treatment sessions and transferring them to everyday life. However, clinical experience shows that patients’ adherence regarding these assignments is often low, thus diminishing the treatment success. One obstacle in this regard is a lack of motivation. The use of smartphone apps in the context of child and adolescent psychotherapy is relatively new, and may provide novel ways to improve the transfer of coping strategies to daily life between treatment sessions. However, only a small number of high-quality studies have analyzed the systematic use of smartphone apps in therapy. The present study will therefore evaluate patients’ homework assignment adherence when using a smartphone app as compared to a paper-and-pencil method.

**Method:**

The study will be conducted as a randomized controlled trial to evaluate the impact of a smartphone app on the adherence to therapy homework assignments (*n* = 35) in the treatment of children with aggressive behavior aged 6–12 years compared to paper-and-pencil homework assignments (*n* = 35).

**Discussion:**

This trial is intended as a pilot study and aims to provide a basis for a subsequent multicenter trial. However, the results may already lead to recommendations for the development and use of mental health-related smartphone apps for children and adolescents with aggressive behavior problems.

**Trial registration:**

Trial registration AUTHARK: German Clinical Trials Register (DRKS) DRKS00015625. Registered on 15th October 2019.

**Supplementary Information:**

The online version contains supplementary material available at 10.1186/s13063-022-06325-6.

## Background

Various interventions have been proven to be effective for the treatment of children and adolescents with oppositional defiant disorder (ODD) and conduct disorder (CD). In particular, studies have demonstrated the effectiveness of child-focused interventions (social competence training), parent-focused interventions (parent management training), combined child-parent interventions, and multimodal or multi-component programs [1, 2].

Child-oriented approaches within cognitive behavioral therapy (CBT) usually include at least one of the following: techniques to reduce or regulate excessive anger; learning social problem-solving strategies; and developing or practicing social skills as an alternative to aggressive behavior [3]. In Germany, two programs have been developed and evaluated for the treatment of children with ODD/CD and peer-related aggression. The Treatment Program for Children with Aggressive Behavior (THAV) [4] is an individualized social competence training program for children aged 6–12 years, with a distinct focus on peer-related aggression. The THAV program has been found to show significant moderate effects on peer-related aggression as rated by parents compared both to a waiting period [5] and to an active control group [6]. The Social Computer-assisted Training for Children with Aggressive Behavior Problems (ScouT) is an individually delivered, computer-assisted social skills training intervention for children with aggressive behavior [7]. The ScouT has likewise been shown to be effective with regard to parent-rated, peer-related aggression compared to a waiting period [8] and to an active control group [9].

However, despite the moderate effects of these interventions, their effects are limited. A major cause of this limited effectiveness may lie in the low adherence to therapy homework assignments aimed at improving the transfer of treatment effects from the therapeutic session to patients’ daily lives. A meta-analysis revealed that the extent to which a patient completed therapy-related assignments between therapy sessions correlated with the treatment outcome [10]. Therapy homework enables patients to apply newly gained social competences in real life. Moreover, the patient’s experiences with these homework assignments are reflected upon during subsequent therapy sessions, thus providing the therapist with important diagnostic information [11, 12].

A study evaluating the treatment processes during the THAV program reported a significant correlation between the total score of a child's adherence (cooperation and implementation of homework) and the reduction in parent-rated oppositional behavior [13]. However, in the evaluation of the ScouT program, no significant correlations were found between child or parental adherence and the reduction in aggressive symptoms [14].

The problem of therapy homework adherence (THA) is not restricted to the treatment of children with externalizing behavior problems, but is also found in the treatment of children and adolescents with other mental disorders [15, 16]. New technologies, such as smartphone apps, may provide novel opportunities to improve THA and may also enhance research options in this field [17].

Smartphone apps can support patients with their therapy homework in everyday life (e.g. arranging to meet up with a classmate) by reminding them of the tasks via push messages or by providing instructions for their implementation. Moreover, they help patients to record real-life experiences using electronic diaries. Reward systems within a smartphone app enable direct feedback on completed tasks, and may consequently also enhance THA [18]. With regard to research options, smartphone apps can provide momentary assessment functions in order to examine current emotional states, cognitions or behavior, and may thus reduce the recall bias [19, 20].

In 2017, Grist et al. [21] identified 15 mental health-related smartphone apps for preadolescents and adolescents which had been described in publications. Most of these apps contained self-observation functions (mood, emotions, behavior), but only one app provided instructions for an active intervention in the form of exposure (The Mayo Clinic Anxiety) [22]. Five of the 15 apps were described as a supplement to face-to-face treatment: Mobile Mood Diary [15], SmartCAT [23], Safety Plan [24, 25], The ACT App [26], an unnamend app [27].

Another systematic review of smartphone-based symptom monitoring and interventions for children, adolescents, and young adults with various mental health disorders identified 15 studies that evaluated a total of 14 different apps [28]. Only eight of these apps included specific treatment content in addition to mood and behavioral monitoring [25, 29–35]. Moreover, only three of the identified studies used a randomized control group design with more than 20 patients within each treatment arm in order to evaluate the effectiveness of an app-augmented treatment compared to a treatment-as-usual intervention group [25, 29, 32].

Furthermore, little research has explored the use and implementation of app content between therapy sessions in greater depth [15, 36]. Although eight of the 15 studies in the systematic review by Melbye et al. [28] reported results on adherence and app acceptance during treatment periods, the studies differed somewhat in how adherence was described (e.g., percentage of tasks completed versus required, achievement of a given skill level). Moreover, adherence usually refers only to the number of tasks completed and not to how users actually perform these tasks [28].

Further studies also generally related app adherence to the number of tasks completed. For example, Kaur et al. [37] found that depressive adolescents used a self-monitoring function of a smartphone app frequently, and in a small pilot study, Mathews & Doherty [15] found an average THA of 65% for completing a daily mood diary.

The SmartCAT app [23] was designed to enable and motivate patients to practice skills outside of therapy sessions. In a small feasibility trial, children and adolescents with anxiety were instructed to use the skills coach when they felt anxious or when they received a reminder from their therapist in the form of a text message. Participants completed an average of 5.36 entries out of 6.48 requests (82.8% completion rate) between each session (SD = 1.95). On average, they spent 3.44 min (SD = 0.98 min) completing the skills coach entries.

To the best of our knowledge, only one study has specifically focused on THA within app-augmented CBT for youth with depression [16]. In an ongoing multisite, randomized controlled pragmatic clinical trial, the authors are evaluating the influence of the app on homework completion. The authors assume that participants who receive the app-augmented treatment will show greater homework compliance. Due to the low compliance rates so far, the app is expected to have moderate effects. For the group comparison across four measurement points, a power of 0.84 was calculated for a sample size of 35 per group (app, no app) to determine effects of homework compliance at α = 0.5. No other results from this study were available for review at the time of submission of this study protocol.

Despite the large number of mental health-related smartphone apps for children and adolescents, only a few have been evaluated in terms of their effectiveness using clinical samples [38]. To date, moreover, there is no methodologically sophisticated study on the use of smartphone apps in the therapy of children with aggressive behavior problems [39, 40], and hardly any consideration has been given to the effects of smartphone apps on therapy homework adherence [18, 41].

Therefore, this pilot study aims to bridge this research gap by evaluating a smartphone-augmented CBT intervention for children with aggressive and oppositional behavior problems with a newly developed app (App-unterstützte Therapiearbeit für Kinder, AUTHARK).

The primary objective of the AUTHARK study is to determine whether participants who receive a smartphone-augmented therapeutic intervention show significantly higher adherence regarding the quantity and quality of completed therapy homework assignments than participants who receive a similar treatment without the support of a smartphone app.

In detail, this study will examine the following hypotheses:

- 1) The experimental group (THAV/ScouT + AUTHARK) will show stronger adherence (implementation of therapy homework assignments (THA)) than the control group (THAV/ScouT), as assessed after each therapy session using the Questionnaire for Therapy Adherence [42] (primary outcome).
- 2) The experimental group (THAV/ScouT + AUTHARK) will show a stronger reduction in aggressive symptoms than the control group (THAV/ScouT) (assessed using the parent-, teacher-, and patient-rated DISYPS-III Symptom Checklist for Disruptive Behavior Disorder (SCL-DBD parent, SCL-DBD teacher, SCL-DBD patient (ages 11–18) [43] and using the individual problem list from the THAV program [4]).
- 3) The experimental group (THAV/ScouT + AUTHARK) will show a stronger reduction in comorbid symptoms than the control group (THAV/ScouT) (assessed using the total score of the German version of the Child Behavior Checklist for ages 6–18 (CBCL/6-18R-total) [44], the total score of the German version of the Teacher’s Report Form for Ages 6–18 (TRF/6-18R-total) [45], the total score of the German version of the Youth Self Report for ages 11–18 (YSR/11-18R-total) [46], and the parent-, teacher-, and patient-rated DISYPS-III Symptom Checklist for Attention-Deficit/Hyperactivity Disorder (SCL-ADHD parent, SCL-ADHD teacher, SCL-ADHD patient (ages 11–18) [43]).
- 4) Patients in the experimental group (THAV/ScouT + AUTHARK) will show a stronger improvement in psychological functions that help to reduce aggressive symptoms (social-cognitive information processing, impulse control, social skills, empathy) than patients in the control group (THAV/ScouT) (assessed using the parent- and patient-rated Questionnaire on Aggressive Behavior in Children (FAVK parent, FAVK patient (ages 9–12) [47], the parent- and patient-rated Inventory of Callous-Unemotional Traits (ICU parent, ICU patient (ages 8–12) [48] [49], and a Social Problem-Solving Test for children ages 6–12 (SPST) [7].
- 5) Patients in the experimental group (THAV/ScouT + AUTHARK) will show a stronger improvement regarding psychosocial functioning and quality of life than patients in the control group (ScouT/THAV) (assessed using the parent-rated modified German version of the Weiss Functional Impairment Rating Scale Parent Report (WFIRS-P-M) [6], the revised parent- and patient-rated Questionnaire for Measuring Health-Related Quality of Life in Children and Adolescents KINDL-R parent, KINDL-R patient (ages 7–13) [50], and the parent- and patient-rated Questionnaire for the Assessment of Adaptive and Maladaptive Emotion Regulation Strategies (FRUST parent, FRUST patient (ages 8–12)) [51].
- 6) Patients and parents/caregivers in the experimental group (ScouT/THAV + AUTHARK) will show higher treatment satisfaction than those in the control group (ScouT/THAV) (assessed using the modified German version of the Client Satisfaction Questionnaire (ZUF-8-M) for parents/caregivers and patients [52]).

A detailed description of the above-mentioned clinical assessment tools is provided in the section “Secondary Outcomes”.

In addition, we will address the following research questions:

1. How feasible is the AUTHARK smartphone app and how satisfied are patients and parents/caregivers with the app?
2. What is the clinical significance of the symptom change in the experimental group compared to the control group?
3. Which moderators/predictors (e.g., gender, age, parental mental health, adverse childhood experiences) can be identified to predict treatment outcome?

## Methods

### Trial design

The AUTHARK study is designed as an interventional randomized controlled study with two parallel treatment arms.

**Fig. 1:**
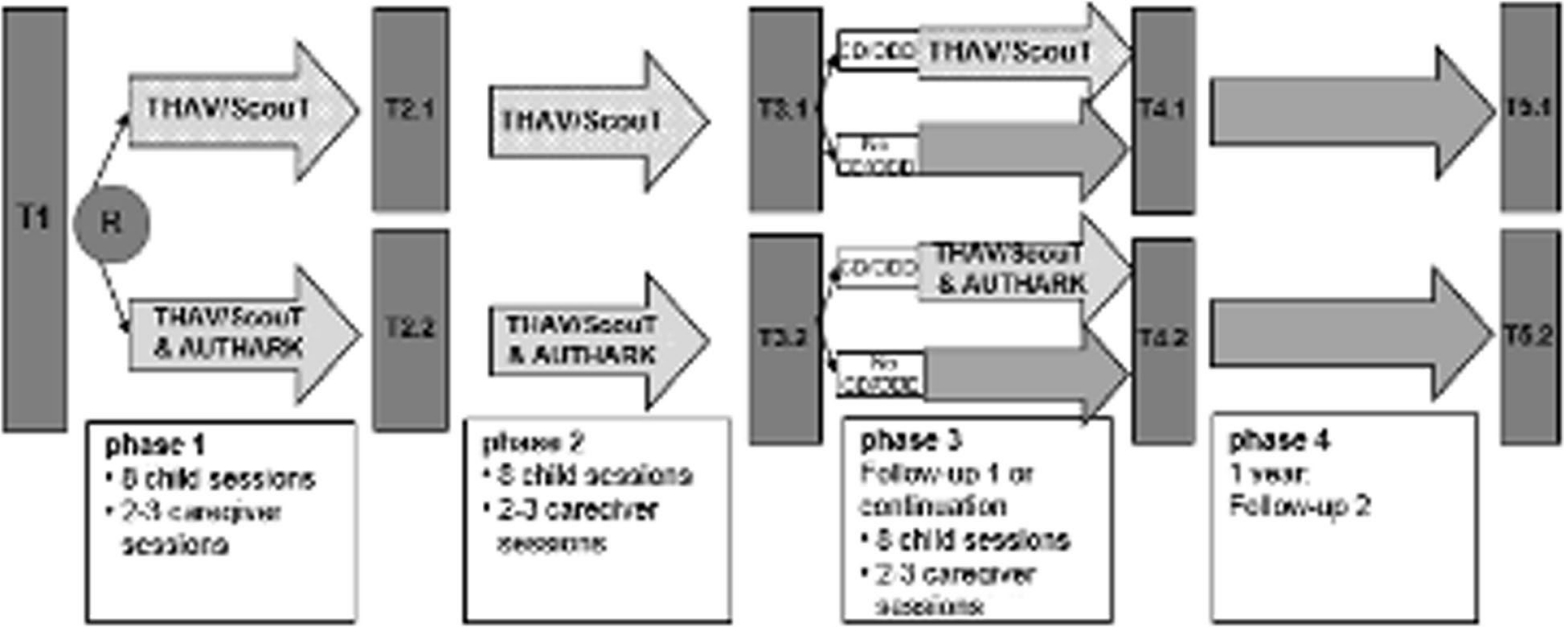
Overall design of the AUTHARK study

Figure 1 shows the overall design of the AUTHARK study. After the initial screening at T1, patients who meet the inclusion criteria will be randomly assigned to one of the treatment arms. Children in both the experimental group and the control group will receive social skills training (combination of THAV [4] and ScouT [7]). In addition to the treatment, the AUHARK smartphone app will be used in the experimental group. The treatment will be administered in three phases (1–3). Each phase includes eight weekly therapy sessions with the children and two to three sessions with the parents/caregivers. The first treatment phase (1) is followed by an intermediate assessment (T2) with a reduced set of assessment instruments. The second treatment phase (2) is followed by a major assessment (T3). Children who no longer show clinically significant symptoms of ODD/CD at T3 will not receive further therapy but will participate in a first follow-up assessment (T4) eight weeks after T3. Those patients with significant symptoms at T3 will undergo a third treatment phase with a further eight child sessions and two to three caregiver sessions, which will be followed by the T4 assessment. A follow-up evaluation (T5) for all patients will take place one year after T4.

### Study setting

The AUTHARK trial is a single-center study located at the School for Child and Adolescent Cognitive Behaviour Therapy (AKiP), Faculty of Medicine and University Hospital Cologne, University of Cologne.

### Recruitment, inclusion and exclusion criteria

Patients for the AUTHARK study will be recruited from three different sites: (1) the outpatient units of the Department of Child and Adolescent Psychiatry and of the School of Child and Adolescent Behaviour Therapy (AKiP) at the University Hospital Cologne, (2) primary and special education schools and (3) pediatric and psychiatric practices in Cologne, Germany.

Participants will be included if they meet the following inclusion criteria: (1) child age: 6;0 to 12;11 years at T1; (2) clinician-rated diagnosis of ODD/CD based on a structured interview (ILF-EXTERNAL) that is part of the comprehensive Structured Interview for Children and Adolescents according to ICD-10 and DSM-5 from the DISYPS Systems (DISYPS- ILF) [53] at T1; (3) parent-rated ODD/CD symptoms from the parent-rated DISYPS III Symptom Checklist for Disruptive Behaviour Disorder (SCL-DBD parent) [43] with a stanine score of 7 or higher at T1; (4) peer-related aggressive (verbal and/or physical) behavior must be reported by the parents/caregivers at T1; (5) willingness and ability (e.g. sufficient knowledge of German, sufficient competence in using a smartphone) of patient and parents/caregivers to participate in the intervention; (6) children treated with a stable dose of psychotropic medication (e.g. methylphenidate) will be included in the trial. Criteria (2) and (3) will also be evaluated again at T3. If these two criteria no longer apply to the patients at T3, the study participation will be terminated.

The following exclusion criteria will be applied and evaluated at T1: (1) child’s intelligence is below average (IQ < 85) based on a standard intelligence test (German version of the Culture Fair Intelligence Test, Primary Intelligence Scale 1 (CFT 1-R) [54] and Scale 2 (CFT 20-R) [55]; (2) Autism spectrum disorder or other severe comorbid disorder which dominates the clinical picture according to clinical rating; (3) Current or planned inpatient psychotherapy or outpatient behavioral parent management training on a weekly/biweekly basis; (4) Low level of German language skills of the patient or the parents/caregivers.

### Allocation

Participants will be randomly assigned to either the treatment condition including the AUTHARK app [56] (*n* = 35) or the active control condition, which includes treatment without the AUTHARK app but with paper-and-pencil homework assignments (*n* = 35). We will use computerized block randomization with a block size of 4 and random selections from all 6 permutations. Allocation will only take place after the participants have completed the initial screening, fulfilled all the inclusion criteria and gave their written consent (patients, parents and teachers). The participants will be randomized by the Principal Investigator AGD. The staff members who are also responsible for the recruitment and the initial diagnostics will request randomization. In return, AGD will send a digital answer form to the same staff member, containing the group allocation (experimental or control group). The staff member will then inform the therapist and the family about the treatment intervention.

Due to the nature of the intervention, it will not be possible to blind trial participants, parents/caregivers, and therapists to allocation. However, the parents/caregivers and patients will be blind regarding the specific hypotheses.

### Interventions

All patients participating in the study will receive behavioral therapy according to German clinical guidelines which recommend the treatment programs THAV and ScouT. The entire duration of the trial will comprise a minimum of 16 child sessions and four parent sessions and a maximum of 24 child sessions and six to nine parent sessions (see “Trial design”). The child sessions will take place on a weekly basis. In both the experimental and control group, participants will be treated with social competence training, consisting of two therapeutic programs: (1) Therapy Program for Children with Aggressive Behaviour (THAV) [4] and (2) Social Computer-Assisted Training for Children with Aggressive Behaviour Problems (ScouT) [7]. Both programs are based on evidence-based international treatment manuals, in particular the Coping Power Program [57] and Cognitive Behavioral Therapy for Anger and Aggression in Children [58].

#### THAV

THAV is a modularized therapy program for children with aggressive behavior aged 6 to 12 years. In a randomized control trial and in a within-subject design, significant moderate effects were found in terms of parent-rated peer-related aggression [5, 6]. The program is organized in five modules with separate components [4]. The modules of the training are: (1) assessment, psychoeducation and development of a therapeutic relationship; (2) social cognitive interventions; (3) anger control training; (4) social problem solving and skills training; and (5) termination and relapse prevention. These five modules are subdivided into a total of 11 treatment units, which are introduced individually as needed. The goals of the social cognitive interventions are to identify and reduce anger-inducing cognitions (*anger thoughts*), identify basic dysfunctional ideas (*thought traps; for example, “I have to be the best of everyone”*), and develop empathy *(“take another perspective!”)*. The anger control training addresses impulse control *(“control your anger!”)*, whereas the purpose of the social problem solving and skills training is to help patients develop and evaluate alternative solutions for a problem situation, and to train them in non-verbal and verbal skillful behavior, including role play*,* video feedback, and role reversal. Self-management, therapy homework assignments, and interventions in the real-life setting *(“Can you manage that in the real world?”)* with self-reinforcement are added in each of these components.

Additionally, a “magic forest game” is used, in which all modules are integrated as a game in which the child has to cope with different tasks which train different aspects of helpful social cognitions, anger control, social cognitive problem solving and behavioral social skills.

In the sessions for parents/caregivers and teachers, the child’s target problems are identified, together with his/her competences and the coercive interaction process. Problem-maintaining social interactions are addressed in each of the modules by interventions that aim to modify these interactions. These interventions include teaching the parents/caregivers how to define social rules, how to communicate effective commands, how to coach the child in social problem situations, how to use methods for rewarding the child (e.g., token systems) when the child shows prosocial behavior, and how to use appropriate methods of punishment (e.g., time-outs) when the child shows aggressive behavior. Additionally, these sessions aim to identify and modify parental dysfunctional thoughts about their child, about their own aggressive behavior, impulse control, and conflict management.

The modularized structure allows for an individual treatment plan, based on the individual difficulties of the patient, e.g. misperception and misinterpretation of social situations, impaired empathy, limited prosocial emotionality or lack of social problem-solving skills. At the beginning of the treatment, an individual behavior problem list is elaborated with the patients’ parents/caregivers. Based on that description and the clinical judgment, an individual treatment plan, consisting of the THAV elements described above, is developed, using a clinical decision-making flowchart [4].

#### ScouT

In Addition to THAV, ScouT will be included in the treatment according to clinical evaluation.

ScouT is an individually delivered, computer-assisted social skills training intervention for children with aggressive behavior aged 6 to 12 years [7]. It combines different cognitive-behavioral methods (e.g., modeling via video sequences and animated cartoon characters, role plays with therapist feedback, homework assignments). The main components are video vignettes of five peer-related conflict situations in which the protagonist is confronted with (1) disappointment, (2) verbal aggression, (3) physical aggression, (4) non-acceptance of responsibility, and (5) depreciation. The work with each video vignette follows a problem-solving approach [59]. The participant (a) first describes the conflict situation shown in the video (including exploring one’s own thoughts, feelings, and actions in such a situation); (b) chooses one of four alternative reactions of the protagonist, which best represents his/her typical reaction (socially competent, socially unassertive, verbally aggressive, physically aggressive); (c) watches a video showing the solution chosen, followed by an exploration of possible thoughts and feelings of both interaction partners and of possible consequences of the behavior, as well as similar conflict situations in his/her daily life; (d) watches a video sequence showing the consequences of the reaction chosen (and of other possible reactions); (e) identifies the best solution for the conflict situation; and (f) transfers it to a real conflict that he/she has experienced in the past.

**Fig. 2:**
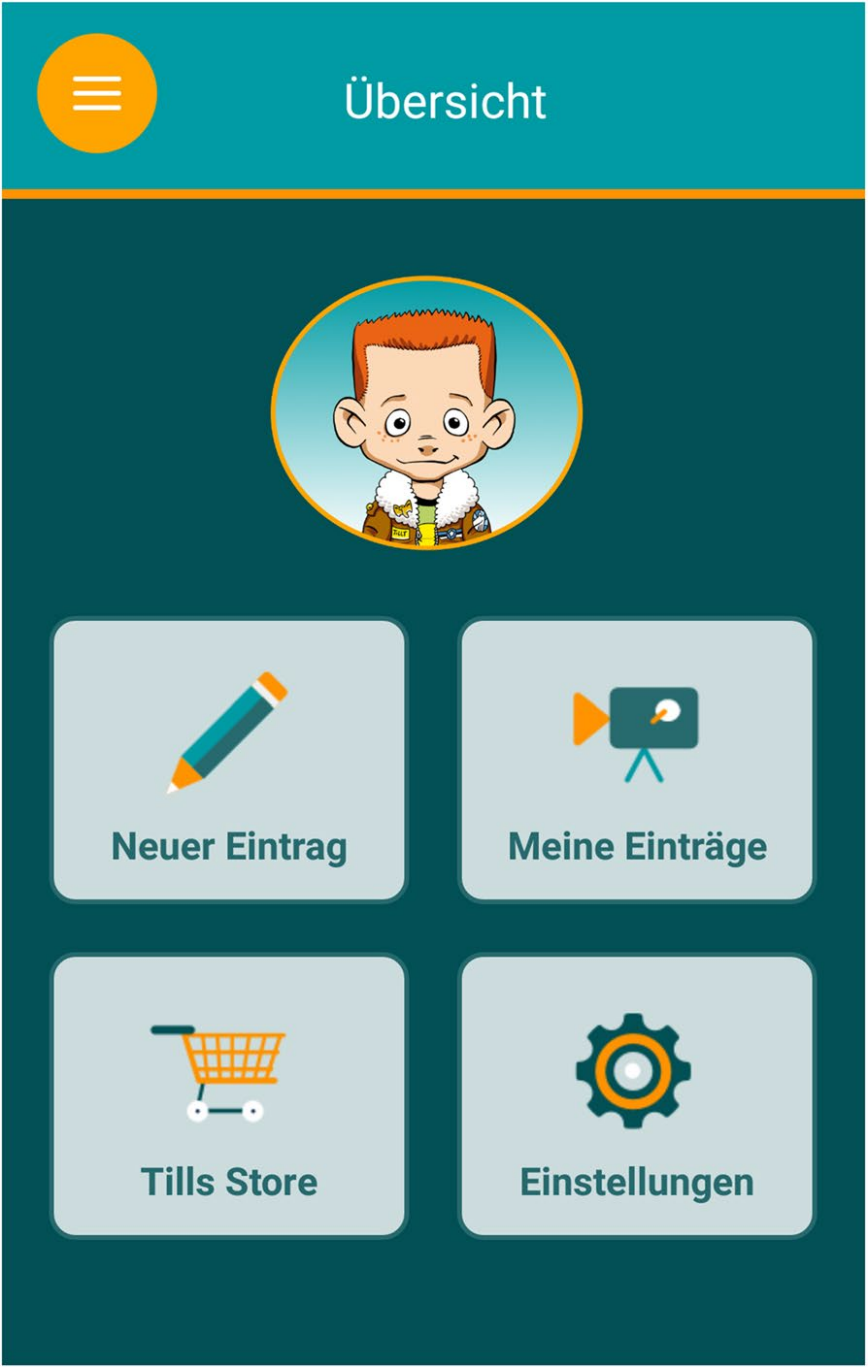
AUTHARK smartphone app (version 2.0), Main navigation [56]

**Fig. 3:**
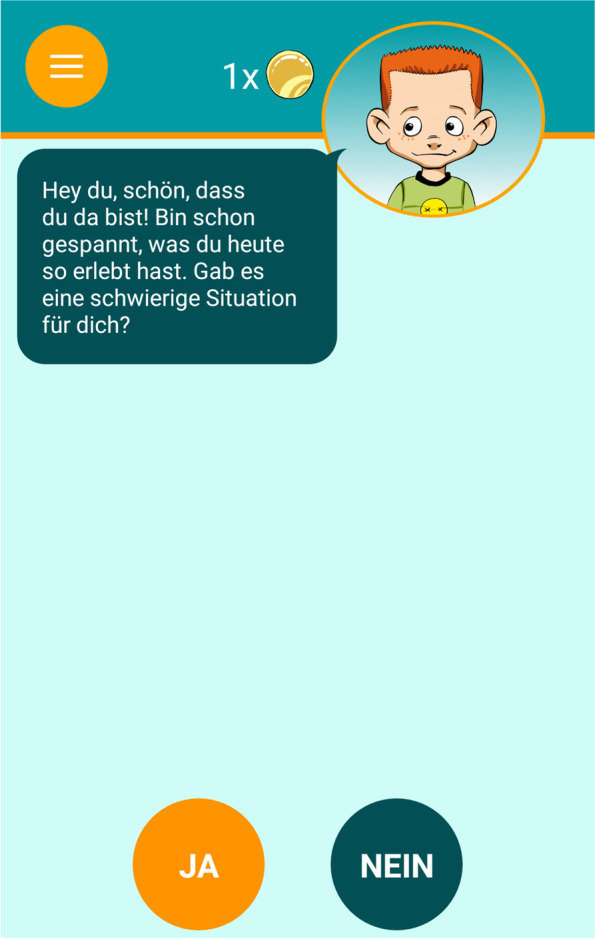
AUTHARK smartphone app (version 2.0), Video diary [56]

**Fig. 4:**
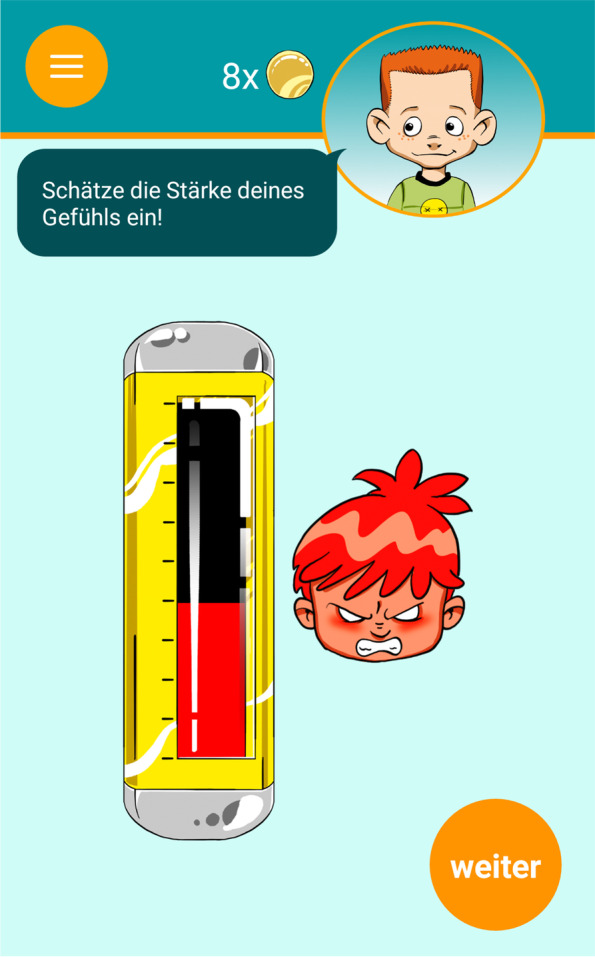
AUTHARK smartphone app (version 2.0), EMA [56]

Therapy homework assignments are an integral part of THAV and ScouT. In the control group, the children and families will complete a paper-and-pencil homework assignment in the form of a self-observation sheet between two therapy sessions. In both groups, homework assignments may comprise reporting on problematic situations in which the child shows aggressive behavior or implementing newly developed strategies during the sessions (Figs. 2, 3 and 4).

### AUTHARK smartphone app

Children and their families in the experimental group will use the AUTHARK app to complete their therapy homework assignments. The AUTHARK app is strongly based on the contents of the THAV and ScouT programs and contains the following functions: (1) a video diary, (2) a momentary assessment function assessing the child’s cognitions, affects and behavior, (3) a training function, (4) a memory function, (5) an information function, and (6) a reinforcement system. The main character from THAV and the ScouT program, Till Taff, also guides the child through the app. For example, he asks the questions in the video diary or asks the child for advice in the training function. The contents of the video diary, the training function and the information function differ according to the patient’s disorders (e.g. depression, ADHD, oppositional and aggressive behavior). These individual settings can be adjusted by the therapist. In this trial, we will focus on oppositional and aggressive behavior. The different functions of the app are described in greater detail below.

The *video diary* contains questions that the child is required to answer through short video recordings. The questions prompt the child to report his / her experiences according to a behavioral analysis (e.g. Did you experience a difficult situation today? What happened? What did you think in this situation? How did you feel in this situation? What did you do or say? What happened afterwards?). If the child has not experienced a difficult situation during the day, he/she is asked about his/her most enjoyable experience of the day in similar manner.

The *momentary assessment function* measures the intensity of basic emotions (happiness, anger, sadness, disgust, and fear). In addition, disorder-specific cognitions, emotions or behaviors can be defined by the therapist and integrated into the assessment. The frequency of the assessment can be adjusted by the therapist.

The *training function* offers the opportunity to practice and improve social skills including problem-solving skills by displaying fictional situations in which the child takes on the role of the expert and helps the character Till Taff in the app to solve a conflict (i.e. gives Till Taff advice).

The *memory function* reminds the child to start his/her therapy homework assignment. Furthermore, the child is asked to report how successful he/she was in implementing the therapy homework using the video diary or, if he/she was unsuccessful, what he/she struggled with.

The *reinforcement function* comes in the form of a virtual store, in which the child can “buy” clothes and accessories for the app’s main character. The coins for the store can be earned within each function.

#### Treatment fidelity

Treatment fidelity will be ensured by (i) training in manualized treatment procedures, (ii) a structured protocol completed by therapists after each session, and (iii) supervision of behavioral therapy by senior supervisors, either face-to-face or by telephone. All treatments will be supervised after every four treatment sessions, including a review of at least two videotaped sessions.

#### Participant timeline

The individual study duration per patient is between five and seven months. Measurements will take place according to a specific schedule. The trial consists of three general parts: a screening process, the intervention period, and the follow-up assessment. Through an initial investigation using standardized telephone screening, a broad assessment of the inclusion and exclusion criteria will be conducted. Shortly after this initial investigation, the pre-treatment measurement (T1) will occur, which involves a detailed examination of the inclusion and exclusion criteria. After being randomized to the experimental or control group, all patients will undergo a first treatment phase (1), consisting of eight patient sessions and two to three parent/caregiver sessions, followed by an intermediate assessment (T2). Subsequently, another treatment phase (2) will be carried out, followed by a main assessment (T3). Non-responders to the treatment (significant symptoms of OD/CDD at T3 based on the clinician-rated parent interview (ILF-EXTERNAL) [53] and on the parent rating (SCL-DBD) from the DISYPS-III [43]) will participate in a third treatment phase (3), followed by a final assessment (T4). Responders to the treatment (no significant symptoms of ODD/CD at T3) will terminate the study and take part in a first follow-up assessment (T3) eight weeks after their last session. After one year, every family will be asked to complete a follow-up survey (T5).

Therefore, four study visits (T1-T4) will take place for all children and parents/caregivers in order to complete assessments. Additionally, there will be 16 study visits for all children and four to six study visits for all parents/caregivers (treatment phases 1 and 2). Within the group of non-responders after T3, there will be a further eight visits for children and two to three visits for parents/caregivers (treatment phase 3).

**Fig. 5:**
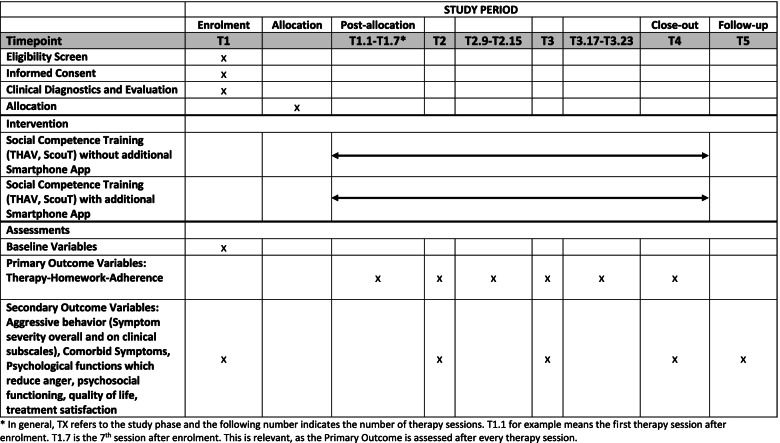
SPIRIT Figure

At the main assessment points (T1, T3/T4), the primary outcome (THA) and the secondary outcomes will be assessed. At the intermediate assessment (T2), the primary outcome and symptoms of ODD/CD as well as comorbid conditions will be assessed using only parent- and child-report questionnaires and without a clinical interview. The primary outcome will additionally be assessed every two weeks, as the therapist will rate the children’s THA using the Adherence Questionnaire (Fig. 5).

#### Informants

The following informants will be considered for assessment: unblinded clinician, parent/caregiver, patient, and teacher. The unblinded clinician will be a member of the project staff who is involved in diagnostics or in therapy (but not in the family being assessed), or will be a child and youth therapist working at the School for Child and Adolescent Cognitive Behaviour Therapy (AKiP). He/she will be aware of the treatment condition and the time of the assessment. The parent may be the biological parent or the child’s guardian and will be involved in the treatment. The patient is the child participating in the study/treatment. The teacher is the child’s schoolteacher, preferably the class teacher with the main responsibility for the child’s school routine. Teachers of patients treated in the control group will remain blinded, as the paper-and-pencil method will not be implemented during school hours.

However, children in the experimental group will be asked to complete an ecological momentary assessment also during school (e.g. at break times). In these cases, teachers may recognize that pupils are in the experimental group.

#### Outcomes

##### Primary outcome

Therapy homework adherence (THA) is defined as the relative ratio of completed to requested entries that are implemented in the video diary or the paper-and-pencil diary (which can be completed either independently by the children or with parental support) between the treatment sessions and the independent overall rating of the quality of entries within an assessment week.

The entire study comprises 24 appointments for the children. Starting at the second therapy session, therapy homework will be assigned every other session (sessions 2, 4, 6, 8, 10, 12, 14, 16, 18, 20, 22, 24). Accordingly, the patients will work on the therapy homework a total of 12 times.

For each assignment, patients will be asked to complete either the video diary or the self-observation sheet on five consecutive school days. The relative ratio can be calculated by dividing the number of completed entries through the number of requested entries. The results will range from 0 to 1.

The independent overall rating of the quality of the video diary or the paper–pencil-diary entries is rated on a 4-point Likert scale (0 = poor quality, 1 = low quality, 2 = largely good quality, 3 = good quality). The overall quality includes two specific criteria: (a) the relevance of the content (spoken or written) to the questions asked, and (b) the level of detail of the spoken or written answers. This rating is based on a rating manual specifying the two quality criteria. Five independent raters will rate the video diary and the paper–pencil diary entries. Interrater agreement will be calculated by conducting intraclass correlation (ICC), using a two-way mixed model for consistency and absolute agreement.

In a preliminary analysis of 15 video diary entries, interrater reliability for five independent raters was conducted. The values for consistency were found to be in the “very good” (0.95) range and the values for absolute agreement were found to be in the “average” (0.73) and “good” (0.87) range [60].

The number of completed entries implemented in the video diary or the paper-and-pencil diary and the overall rating of the quality is assessed trough the newly developed Adherence Questionnaire, which is completed by the therapists after every therapy session, but especially in every session that follows a therapy homework week. For example, when therapy homework is assigned in session 2, the therapist will evaluate the results in session 3.

For the Primary Outcome, we use both aspects of THA described above and thus include quantitative and qualitative features. To determine a THA Score we calculate the product of the relative ratio (e.g. three completed entries with five required entries (3/5 = 0.6) and the overall quality score (e.g. 2). The THA Score ranges between 0 and 3.

##### Secondary outcomes

As a secondary Outcome, we will analyze the quantity (relative ratio) and the overall quality rating separately. Additionally, the percentage of tokens received from the therapists for homework compliance will be considered as a secondary outcome measure.

Further secondary outcomes listed below will be assessed at T1 (baseline) and at T3 or T4:

1. Parent-, teacher-, and patient-rated symptoms of ODD/CD, which will be assessed using the respective rating scales: the parent-, teacher-, and patient-rated DISYPS-III Symptom Checklist for Disruptive Behavior Disorder (SCL-DBD parent, SCL-DBD teacher, SCL-DBD patient (ages 11–18)) [43]. Reliability and validity as well as the sensitivity to change of these scales have already been proven in German samples [43].
2. Clinician-rated symptoms of ODD/CD using the Structured Interview for Disruptive Behavior Disorder (ILF-EXTERNAL), which is part of the comprehensive Structured Interview for Children and Adolescents according to ICD-10 and DSM-5 from the DISYPS-Systems (DISYPS-ILF) [53].
3. Overall behavioral and emotional problems assessed using the total score of the German version of the Child Behavior Checklist for ages 6–18 (CBCL/6-18R-total) [44], the total score of the German version of the Teacher’s Report Form for ages 6–18 (TRF/6-18R-total) [45], and the total score of the German version of the Youth Self Report for ages 11–18 (YSR/11-18R-total) [46]. Reliability and validity as well the sensitivity to change of the German versions of these scales have been demonstrated in several studies [44].
4. Triggering and maintaining components of aggressive behavior (social-cognitive information processing, impulse control, social skills, and reactions to the social environment) assessed using the parent- and patient-rated Questionnaire on Aggressive Behavior in Children (FAVK parent, FAVK patient (ages 9–12) [47].
5. Parent-rated satisfaction with the treatment, assessed using the modified German version of the Client Satisfaction Questionnaire (ZUF-8-M parent), and patient-rated satisfaction with the treatment, assessed using the modified German version of the Client Satisfaction Questionnaire (ZUF-8-M patient) [61], which has been shown to be reliable, valid, and sensitive to change [62].

##### Supplementary outcomes

The supplementary outcomes listed below will be measured (pre- and post-treatment) to assess the potential benefit for the main study:

1. Parent-, teacher-, and patient-rated symptoms of ADHD, which will be assessed using the respective rating scales: the parent-, teacher-, and patient-rated DISYPS-III Symptom Checklist for Attention-Deficit/Hyperactivity Disorder (SCL-ADHD parent, SCL-ADHD teacher, SCL-ADHD patient (ages 11–18) [43]. Reliability and validity as well as the sensitivity to change of these scales have already been proven in German samples [43].
2. Individually defined problem behaviors assessed with the parent-rated Individual Problem Checklist (IPC) taken from the THAV manual [4]. The IPC captures four individual parent-defined target problems to be rated on a 6-point Likert-type scale, and has been shown to be reliable, valid, and sensitive to change [63].
3. Patients’ adherence during the weekly therapy sessions (e.g. punctuality, participation in assignments and activities) and parents’/caregivers’ adherence during a parent appointment, assessed using the clinician-rated Adherence Questionnaire [42], which was specifically developed for this trial and is based on items from an already existing questionnaire on treatment adherence which showed good internal consistencies (child: α = 0.90 to α = 0.95; parent: α = 0.88 to α = 0.97) [13].
4. Parents’/caregivers’ adherence regarding possible assignments between the child’s therapy sessions or between the parent/caregiver appointments, assessed using the clinician-rated Adherence Questionnaire (see above).
5. Callous-unemotional traits in children (e.g. lack of empathy) assessed using the parent- and patient-rated German version of the Inventory of Callous-Unemotional Traits (ICU parent, ICU patient (ages 8–12) [48] [49], which has been shown to be reliable, valid, and sensitive to change [64].
6. Problem-solving competences and social information processing assessed using the computer-based Social Problem-Solving Test for children aged 6–12 (SPST), which has been shown to be reliable and valid [7].
7. Strategies for the regulation of emotions assessed using the parent- and patient-rated Questionnaire for the Assessment of Adaptive and Maladaptive Emotion Regulation Strategies (FRUST parent, FRUST patient (ages 8–12) [51], which is based on the German Questionnaire for the Assessment of Emotion Regulation in Children and Adolescents [51].
8. Children’s health-related quality of life measured using the revised parent- and patient-rated Questionnaire for Measuring Health-Related Quality of Life in Children and Adolescents (KINDL-R parent, KINDL-R patient (ages 7–13)) [50], assessing factors regarding the child’s self-esteem, family, friends, physical health, everyday life, and school as aspects of well-being.
9. Parent-rated psychosocial impairment of the child assessed using the modified German version of the Weiss Functional Impairment Rating Scale Parent Report (WFIRS-P-M) [6] (original version: WFIRS-P [65, 66]), which has been shown to be reliable, valid, and sensitive to change [67].
10. Self-reported parental aggression, anxiety, depression, and stress assessed using a modified short version of the German Questionnaire on Parental Aggression (FB-Ä) [68] and the German version of the Depression Anxiety Stress Scale (DASS) [69], which has been shown to be reliable, valid, and sensitive to change [70].

##### Predictors/moderators of treatment outcome regarding ODD/CD symptoms and impairment

As moderators or predictors of treatment outcome at T4 regarding ODD/CD symptoms and impairment, the following variables will be analyzed: (1) sociodemographic variables (e.g. place of residence, socioeconomic status; education, income, occupation and age of parents/caregivers; number of children in the family), which will be assessed through parent/caregiver interview at T1, (2) severity of comorbid disorders at T1-T3 using the patient- and parent-rated SCL-ADHD, the parent-rated CBCL/6-18R, and the teacher-rated TRF/6-18R (see above), (3) specific psychological traits of the parents/caregivers (parental aggression, depression and stress) at T1 and T3 using the FB-Ä and DASS (see above), (4) specific psychological traits of the patient (intelligence), assessed at T1 using the German version of the Culture Fair Intelligence Test, Primary Intelligence Scale 1 (CFT 1-R) [54] and Scale 2 (CFT 20-R) [55].

##### Mediators of change

As one potential mediator, (1) treatment fidelity will be assessed using an adapted version of the THAV Integrity Questionnaire [71] and the ScouT Treatment Integrity Questionnaire [72], which measure treatment integrity throughout the whole treatment process. After each unit of THAV/ ScouT therapy, the therapist will record the percentage of time he/she spent implementing the specific therapy elements from the THAV or ScouT program. In addition, the therapist will document which materials (e.g., worksheets, therapeutic stories, etc.) were used during the treatment sessions. The THAV Integrity Questionnaire has shown satisfactory reliability scores [13] and the ScouT Treatment Integrity Questionnaire has shown excellent internal consistencies [14]. Moreover, as a possible mediator, we will measure the (2) therapist-patient relationship and therapist-caregiver relationship using the German Relationship Questionnaire for Child and Adolescent Psychotherapy (BeKi) [73]. In order to assess each alliance from the perspectives of both parties, the BeKi has four separate versions: two for the therapist evaluating his/her alliance to the patient (BeKi therapist-patient) or parent (BeKi therapist-parent), and one each for the patient (BeKi patient) and the parent (BeKi parent) evaluating their respective alliances to the therapist.

(3) Moreover, we will assess the parent rating of adaptive and maladaptive emotion regulation strategies (FRUST parents, see above) [51].

(4) Additionally, after every therapy session, we will assess treatment adherence as a mediator using the Adherence Questionnaire. Four different items will be used to operationalize adherence as the child’s cooperation during the therapy session and adherence as the implementation of therapy homework. For this study, a modified version of the Adherence Questionnaire [42] will be used. Internal consistencies of the original Adherence Questionnaire range between α ≥ 0.95 or α ≥ 0.95 for children and α ≥ 0.97 or α ≥ 0.97 for parents [13, 14].

#### Sample size

A total number of 70 children 35 / 35 between the ages of 6;0 and 12;11 will be included in this exploratory study. To date, the effects of smartphone-augmented CBT on the implementation of therapy homework in children with aggressive behavior have barely been investigated. Other explanatory studies refer to an even a smaller number of cases [15, 23]. As the AUTHARK study aims to provide a basis for preparing a subsequent multicenter trial, the sample size needed is not based on formal power calculations. Instead, a sample size is proposed that allows us to precisely estimate the variance of critical outcome parameters that can then be entered into a power calculation for a confirmatory trial. In this way, the overall sample size for the main RCT can be minimized [74].

Based on results from the previous literature, we assume a small to moderate effect size (Cohen’s d ~ 0.4) [75], for which approximately 30 participants per treatment arm for a pilot randomized trial are recommended [74, 76]. Furthermore, a study with a similar design, comparing two groups across four assessment points (pre-, post-, and follow-up measurements), expects moderate effects regarding the app’s influence on therapy homework with a sample size of *n* = 35 per group [16]. Moreover, the power of a sample can be increased by a high number of measurements per patient [77]. In our study, THA will be measured at least eight times over a period of approximately sixteen weeks. At each assessment, the patients will be asked to use the smartphone app for five consecutive schooldays. Based on previous trials with a similar patient population and interventions, a dropout rate between 10 and 15% is expected [78]. Therefore, *n* = 35 patients per group (total: *n* = 70) should be recruited.

#### Data management and confidentiality

Most of the trial data will be assessed electronically through an online survey tool (LimeSurvey) [79]. After the completion of an assessment point, the stored data will be exported from the platform by a designated employee and saved in an encrypted data network. For each assessment point, the data will be organized according to patient-, caregiver-, and teacher ratings (see above).

The primary outcome of the trial, the THA, will be assessed weekly using the Adherence Questionnaire (see above), which will be completed by the therapists in a paper-and-pencil form in both groups. The data will then be re-entered digitally by one designated employee. The data will be regularly reviewed for completeness, consistency, and plausibility by two members of the project. After each assessment week, the data from the AUTHARK smartphone app (video diary, momentary assessment function) will be exported, transferred and saved to an encrypted network, to which only selected project members will have access.

At the beginning of the study, all participants will receive an individual study identification number. Data collected within the study will be anonymized and can only be associated with the patient by the project staff through the study identification number. Legal regulations for data protection will be fulfilled. All participating families and teachers will be informed about their rights in terms of data storage, data protection, and data deletion.

Informed consent will be obtained both in both written and verbal form from patients, parents/caregivers or guardians, and (if applicable) teachers before screening. For detailed information, see Additional file 2: Model written informed consent. All participants will be able to clarify questions about the study with research staff members. To participate in the study, it is required that both the patient and his/her parents/caregivers or guardians give their assent and informed consent. Likewise, teachers must give their informed consent for study participation. Data entry and processing will take place as soon as signed informed consent is available.

This study is designed as a small monocenter pilot study with no external funding, and there will be no external data monitoring or auditing committee, although this will be considered for a subsequent larger trial.

#### Statistical analyses

##### Patient demographics/other baseline characteristics

Demographic and other baseline data will be obtained at T1 and will be summarized descriptively for all documented patients. Continuous data will be summarized by arithmetic mean, standard deviation, minimum, 25% quantile, median, 75% quantile, maximum, and the number of complete and missing observations. If appropriate, continuous variables can also be presented in categories. Categorical data will be summarized according to the total number of patients in each category and the number of missing values. Relative frequencies will be displayed as valid % (number of patients divided by the number of patients with non-missing values).

##### Analyses of primary endpoint

The primary (full) analysis set is derived from the intention-to-treat (ITT) principle (all patients randomized with a valid baseline assessment). Additionally, per protocol analyses will be conducted based on all patients with at least 50% of the planned treatment sessions and a valid follow-up. As primary endpoint, the overall scores on THA will be calculated from the baseline, intermediate, and final assessment points (T1, T2, T3). THA scores assessed after each session will also be included in the analyses. A mixed model repeated measures multivariate analysis of variance will be performed with the between factor Group (smartphone-augmented vs. traditional), the within factor Time (baseline, first treatment phase, intermediate assessment, second treatment phase, final assessment) and the two-way interaction of Group x Time.

##### Analyses of secondary endpoints

Secondary outcomes will be analyzed using either mixed models for repeated measures (heterogeneous first-order autoregressive structured covariance matrix over time) or generalized estimating equation approaches with corresponding marginal means and contrast tests (multilevel modeling). Time-to-dropout distributions will be summarized by the Kaplan–Meier method and compared using the (stratified) log-rank test. All efficacy variables will be summarized by time point and treatment arm (mean, standard deviation, percentiles (i.e., minimum, 25^th^, 50^th^, 75^th^, and maximum), count, percentage). Moreover, moderation, mediation and conditional process modeling [80] will be conducted based on regression and structural equations (interaction, simple slope analysis; direct/indirect effects, kappa squared).

##### Missing values

It should be emphasized that as few patients as possible should discontinue treatment and that all patients should be followed up and documented after discontinuation of the treatment in order to record data required according to the ITT principle. To assess the impact of up to 20% attrition, multiple imputation approaches will be taken, accounting for proxy measures and assuming specific missingness-not-at-random patterns. Sensitivity analysis with a data set with and without imputation will be computed to assess the effects of missing values. The details will be documented in a statistical analysis plan. The analysis of subjects essentially observed and treated per protocol is supportive.

#### Stopping rules

##### Stopping rules for an individual patient

One (or more) of the following circumstances will result in an early study termination of single subjects (these trial subjects will be rated as dropouts): (i) withdrawal of informed consent of one parent (in the case of joint custody)/caregiver/guardian; (ii) withdrawal of assent of the patient; (iii) unwillingness to further participate in the trial; (iv) need for inpatient treatment or other reasons affecting the patient’s well-being in the case of continued trial participation; (v) need for a different kind of treatment for health reasons according to the judgment of the attending physician.

##### Global stopping rules

A termination of the entire trial will be executed if less than 50% of the planned sample size is recruited despite additional recruitment strategies. This decision will be made by the Principal Investigators.

## Discussion

The AUTHARK study will yield information about the effects of smartphone-augmented CBT on the THA compared to conventional paper-and-pencil assignments completed between therapy sessions for children aged 6–12 years with a diagnosis of ODD/CD.

The study will also explore the role of therapy homework as a possible mediator of treatment outcome. Furthermore, the trial will describe possible effects of app-augmented CBT on the symptoms of ODD / CD, ADHD, and other comorbid behavioral or emotional problems; on individually defined problem behaviors; on triggering and maintaining factors of peer- and adult-related aggression (social cognitive information processing, impulse control, social skills, empathy, adaptive and maladaptive strategies for regulation of emotions, problem solving competences); on the children’s quality of life; on the children’s and parents’/caregivers’ level of psychosocial functioning; and on the parents’/caregivers’ and patients’ satisfaction with the treatment.

Since this study is designed as a pilot study, there are some aspects that may prove challenging during the course of the study. One of these aspects is the young age of the children we plan to include. However, our preliminary experience with children in this age range is that even children aged six to seven years are able to handle the smartphones and the requirements of the app. This experience is also supported by the results of a small pilot study that investigated the use of a language app with kindergarten children. The authors reported that children aged three to five years were able to use the app well with support [81]. Moreover, the effects of age on usability and adherence to the intervention will be analyzed.

Another challenge of the study is the risk of bias regarding the outcome ratings. Although participating families will not be informed about the hypotheses of the study, they may conclude from the information letter that the use of the AUTHARK app is an innovation and is expected to result in higher therapy homework adherence. Likewise, the treating therapists cannot be blinded to the intervention. However, the detailed descriptions of quality criteria, on which the rating is based, (see “15”) may reduce the risk of bias. Moreover, in a subsequent main study, an independent blinded evaluation of therapy homework adherence will be considered.

Nevertheless, the results will have an impact on clinical practice, as they will demonstrate how and to what extent a smartphone app can be used for diagnosis and intervention within CBT with children. Furthermore, the results will demonstrate how the implementation of assignments between therapy sessions can be improved. Finally, our findings will indicate the relevance of various dimensions for the therapy outcome.

The results of the AUTHARK study can contribute to an improvement in the development of apps in child and adolescent psychotherapy and will offer some new options in the treatment of aggressive behavior in children.

### Public or patient involvement

The protocol was written based on the study design by AGD. There was no public or patient involvement in the planning of the trial or in the process of writing this study protocol.

### Trial status

#### Protocol version: 01 / 2022, issue date: 06.04.2022

The recruitment for the AUTHARK study has already started in October 2019 and is still ongoing by the time of the submission of this protocol. The completion of recruitment is planned for May 2022. Due to the Covid-19 pandemic and its various challenges regarding ongoing research projects, this paper could not be finalized and submitted earlier.

## Supplementary Information

**Additional file 1: **Trial registration AUTHARK.

**Additional file 2: **Model written informed consent.

## Data Availability

After publication of the results, the final datasets can be obtained from the Principal Investigator (Anja Görtz-Dorten). The results of study, especially regarding the primary outcome, will be submitted for publication in relevant journals. Furthermore, patients and their families will have access to the results if they wish. The results will also be presented at internal meetings and workshops as well as at relevant congresses.

## Abbreviations

ADHD: Attention-deficit/hyperactivity disorder
AKIP: Outpatient unit of the Department of Child and Adolescent Psychiatry and of the School of Child and Adolescent Behaviour Therapy
AUTHARK: App-augmented therapeutic treatment for children with aggressive behaviors
BeKi: German Relationship Questionnaire for Child and Adolescent Psychotherapy
CBCL/6-18R: German version of the Child Behavior Checklist for ages 6–18 (parent-rated)
CBT: Cognitive Behavioral Therapy
CFT 1-R: German version of the Culture Fair Intelligence Test, Primary Intelligence Scale 1
CFT 20-R: German version of the Culture Fair Intelligence Test, Primary Intelligence Scale 2
CD: Conduct disorder
DASS: German version of the Depression Anxiety Stress Scale
DISYPS-III: Diagnostic System for Mental Disorders in Children and Adolescents
DISYPS-ILF: The comprehensive Structured Interview for Children and Adolescents according to ICD-10 and DSM-5 from the DISYPS-Systems
DRKS: Deutsches Register Klinischer Studien (German clinical trials register)
DSM-5: Diagnostic and statistical manual of mental disorders of the American Psychiatric Association, 5th edition
FB-Ä: Modified short version of the German Questionnaire on Parental Aggression
FAVK: Parent- and patient- (ages 9–12) rated Questionnaire on Aggressive Behavior in Children
FRUST: Parent- and patient- (ages 9–12) rated Questionnaire for the Assessment of Adaptive and Maladaptive Emotion Regulation strategies
ICD-10: International statistical classification of diseases and related health problems, 10th revision
ICU: Parent- and patient-rated Inventory of Callous-Unemotional Traits
ID: Identification number
ILF-External: Structured interview for the therapist’s rating of externalizing symptoms
IPC: Individual Problem Checklist
ITT: Intention-to-treat
KINDL-R: Revised parent- and patient- (ages 7–13) rated Questionnaire for Measuring Health-Related Quality of Life in Children and Adolescents
ODD: Oppositional defiant disorder
RCT: Randomized controlled trial
SCL-ADHD: Parent-, teacher-, and patient- (ages 11–18) rated DISYPS-III Symptom Checklist for Attention-Deficit/Hyperactivity Disorder
SCL-DBD: Parent-, teacher-, and patient- (ages 11–18) rated DISYPS-III Symptom Checklist for Disruptive Behaviour Disorder
ScouT: Social Computer-Assisted Training for Children with Aggressive Behaviour Problems
SPST: Social Problem-Solving Test for children ages 6–12 from the ScouT program
THA: Therapy homework adherence
THAV: Treatment Program for Children with Aggressive Behaviour Problems
TRF/6- 18R: German version of the Teacher’s Report Form for Ages 6–18
WFRIS: Weiss Functional Impairment Rating Scale
WFRIS-P-M: Modified German version of the Weiss Functional Impairment Rating Scale Parent Report
YSR/11-18R: German version of the Youth Self Report for ages 11–18
ZUF-8: German version of the parent- and patient-rated Client Satisfaction Questionnaire
ZUF-8-M: Modified German version of the parent- and patient-rated Client Satisfaction Questionnaire
