# Supplementary material for: Effects of a smartphone app-augmented treatment for children with oppositional defiant disorder / conduct disorder and peer-related aggressive behavior – a pilot study
Source: Trials. 2022 Jul 8;23:554. doi: 10.1186/s13063-022-06325-6 (PMC9264298; doi:10.1186/s13063-022-06325-6)
Supplement: Supplementary file 1 — Additional file 1: Trial registration AUTHARK. [file 13063_2022_6325_MOESM1_ESM.pdf]

Vorschau der Studiendaten gedruckt am 09. June 2021 12:40:38

DRKS-ID der Studie: **DRKS00015625**

Studienbeschreibung

Titel der Studie

Evaluation einer app-unterstützten Therapiearbeit (AUTHARK) in Kombination mit dem multimodalen Therapieprogramm zur Behandlung von Kindern mit aggressiven Verhalten (THAV) und dem sozialen computergestützten Training (ScouT) im Vergleich zu einer Behandlung mit den Therapieprogrammen THAV und ScouT ohne Unterstützung durch eine Smartphone-App.

Studienakronym

AUTHARK

Internetseite der Studie

http://uk-koeln.de/authark

Allgemeinverständliche Kurzbeschreibung

Am Ausbildungsinstitut für Kinder- und Jugendlichenpsychotherapie der Uniklinik Köln wurden das Therapieprogramm für Kinder mit aggressivem Verhalten (THAV; Görtz-Dorten & Döpfner, 2019) und das soziale computergestützte Training (ScouT; Görtz-Dorten & Döpfner, 2016) entwickelt. Sie richten sich an Kinder im Alter von sechs bis zwölf Jahren. Die Schwerpunkte beider Programme liegen auf der Schulung der sozialkognitiven Informationsverarbeitung, der Entwicklung und Stärkung von Impulskontrolle, dem sozialen Kompetenztraining sowie auf der Modifikation sozialer Interaktionen. Es gibt bereits Voruntersuchungen, die den Programmen eine hohe Wirksamkeit bescheinigen.
Die vorliegende Studie untersucht die zusätzlichen Effekte der therapieunterstützenden App (AUTHARK; Görtz-Dorten & Döpfner; 2019) bei Kindern, die mit den Programmen THAV und ScouT behandelt werden. Die therapieunterstützende App baut auf THAV und ScouT auf. Sie enthält Funktionen, die den Übertrag von Therapieinhalten in den Alltag erleichtern, aber auch Alltagssituationen für die Therapie leichter zugänglich machen sollen. Zu diesen Funktionen zählen das Führen eines Videotagebuchs, eine Stimmungs- und Verhaltensabfrage (Momentary-Assessment), die Erinnerung an individuell vereinbarte Therapieaufgaben, sowie eine Trainingsfunktion der erarbeiteten Inhalte.
Es soll überprüft werden, ob die Kombinationsbehandlung mit der App dazu führt, dass die Kinder die Therapieaufgaben im Alltag besser umsetzen können. Weiterhin soll die Studie prüfen, ob die zusätzliche Nutzung der App während der Therapie, das aggressive Problemverhalten oder auch andere problematische Verhaltensweisen stärker verringert, als eine Therapie ohne die unterstützende App.
An der Studie nehmen 60 Kinder zwischen 6 und 12 Jahren mit aggressivem Verhalten gegenüber Gleichaltrigen teil. Zunächst durchlaufen alle Kinder eine ausführliche Eingangsuntersuchung. Hierzu werden Fragebogenverfahren angewendet und persönliche Interviews geführt. Dann werden die Kinder nach dem Zufallsprinzip in zwei gleich große Gruppen aufgeteilt.
Die eine Hälfte der Kinder (50%) wird mit den Therapieprogrammen THAV & ScouT behandelt. Die zweite Hälfte der Kinder (50%) wird mit den Therapieprogrammen THAV & ScouT, sowie der therapieunterstützenden App AUTHARK behandelt.
Es finden wöchentliche Therapiesitzungen statt. Während der Therapie wird alle zwei Wochen ein Beobachtungsbogen, bzw. Videotagebuch geführt. Zusätzlich werden zu drei festgelegten Zeitpunkten weitere Fragebogen ausgefüllt. Anhand dieser Bogen kann die Umsetzung der Therapieaufgaben, der Verlauf der Probleme während der Therapie und die Wirksamkeit der Behandlung überprüft werden.
Die Gruppe, die zusätzlich mit der App AUTHARK behandelt wird, bekommt hierfür über einen mit der Familie gemeinsam definierten Zeitraum ein Smartphone zur Verfügung gestellt.
Die Therapie innerhalb der Studie umfasst 16 bis 24 Patient*innenkontakte, zuzüglich maximal zwölf Bezugspersonen-Termine. Das heißt im Regelfall beträgt die Gesamtdauer der Teilnahme mindestens 24 Wochen.
Teilnehmen können Familien, deren Kinder zwischen 6 und 12 Jahre alt sind und aggressives Verhalten gegenüber Gleichaltrigen zeigen. Außerdem müssen weitere Bedingungen für die Studiendurchführung

erfüllt sein (z.B. ausreichende Kenntnisse der deutschen Sprache bei Kindern und Eltern).

Wissenschaftliche Kurzbeschreibung

Am Ausbildungsinstitut für Kinder- und Jugendlichenpsychotherapie der Uniklinik Köln wurden zwei Therapieprogramme für Kinder von sechs bis zwölf Jahren mit aggressiven Verhaltensauffälligkeiten entwickelt: THAV und ScouT.
THAV und ScouT sind der multimodalen Psychotherapie verpflichtet, die patient*innen- und umfeld-zentrierte Interventionen miteinander verbindet und damit den Leitlinien zur Therapie von Kindern mit aggressivem Verhalten entspricht. Bei diesen beiden Therapieprogrammen kommen kognitive und behaviorale Behandlungstechniken zum Einsatz, die sich in der Forschung als sehr wirksam erwiesen und in der Praxis bewährt haben. Die patient*innenzentrierten Interventionen setzten in Situationen an, in denen das Kind aggressives Verhalten gegenüber Gleichaltrigen zeigt. Die Schwerpunkte liegen hierbei auf der Schulung der sozialkognitiven Informationsverarbeitung, der Entwicklung und Stärkung von Impulskontrolle, dem sozialen Kompetenztraining sowie auf der Modifikation sozialer Interaktionen. Darüber hinaus beziehen die Therapieprogramme auch familien- und schulzentrierte Interventionen mit ein. Die Wirksamkeit von THAV und ScouT wurde in eigenen Studien belegt (Goertz-Dorten et al. 2017, Goertz-Dorten et al. 2018a; Goertz-Dorten et al., 2018b; Goertz-Dorten et al., accepted; Lindenschmidt, 2016).
Ziel dieser Untersuchung ist die Evaluation einer eigens entwickelten Therapie-App bei Kindern mit aggressiven Verhalten (AUTHARK).
Die App, die auf den Therapieprogrammen THAV und ScouT basiert, enthält folgende Funktionen: Die Stimmungs- und Verhaltensabfrage, die Videotagebuchfunktion, Erinnerung an die Therapieaufgabe und die Trainingsfunktion. Damit können spezifische Informationen über vom Kind erlebte Konfliktsituationen und Emotionen im Alltag erfasst und die Durchführung von Therapieaufgaben unterstützt werden. So kann der Transfer von Bewältigungsstrategien, die in der Therapie erarbeitetet wurden, in den Alltag der Kinder unterstützt und überprüft werden. Weiterhin soll die Motivation zur Durchführung der Therapieaufgaben erhöht werden.
Ziel des Gesamtprojektes ist der Vergleich von den Effekten einer Behandlung mit den Therapieprogrammen THAV und SCOUT kombiniert mit AUTHARK und einer Behandlung mit den beiden Therapieprogrammen ohne eine zusätzliche App-Unterstützung.
Dazu werden folgende inhaltliche Hypothesen aufgestellt, die im randomisierten Kontrollgruppen-Design überprüft werden.
1.) In der Experimentalgruppe THAV/ScouT+AUTHARK zeigt sich eine stärkere Verbesserung der Compliance (Umsetzung von Therapieaufgaben) als in der Kontrollgruppe THAV/ScouT (erfasst über FB Compliance, nach jeder Sitzung). (primäre Zielgröße)
2.) In der Experimentalgruppe THAV/ScouT+AUTHARK zeigt sich eine stärkere Reduktion der aggressiven Symptomatik als in der Kontrollgruppe THAV/ScouT (erfasst über FBB-SSV-Eltern, FBB-SSV-Lehrer, SBB-SSV, individuelle Problemliste)
3.) In der Experimentalgruppe THAV/ScouT+AUTHARK zeigt sich eine stärkere Reduktion der komorbiden Symptomatik als in der Kontrollgruppe THAV/ScouT (erfasst über CBCL-Total, TRF-Total, YSR-Total, FBB-ADHS-Eltern, FBB-ADHS-Lehrer, SBB-ADHS, DADYS-E/K und FRUST-F/S)
4.) In der Experimentalgruppe THAV/ScouT+AUTHARK zeigt sich eine stärkere Verbesserung der psychischen Funktionen der Patient*innen, die zur Entwicklung der aggressiven Symptomatik beitragen (sozial-kognitive Informationsverarbeitung, Impulskontrolle, soziale Fertigkeiten, Empathie) als in der Kontrollgruppe THAV/ScouT (erfasst über FAVK- F/ FAVK-S, ICU-E/K, ScouT-Diagnostik)
5.) In der Experimentalgruppe THAV/ScouT+AUTHARK zeigt sich eine stärkere Verbesserung des psychosozialen Funktionsniveaus und der Lebensqualität der Patient*innen als in der Kontrollgruppe ScouT/THAV (erfasst über die Skala zum Funktionsniveau, KINDL-R)
6.) In der Experimentalgruppe ScouT/THAV+AUTHARK zeigt sich eine stärkere Zufriedenheit der Patient*innen, der Eltern und der Therapeut*innen als in der Kontrollgruppe ScouT/THAV (erfasst über CSQ)

Selbst vergebene Schlagwörter

Verhaltenstherapie für Kinder, Aggressives Verhalten, Evaluationsstudie, Smartphone gestützte Verhaltenstherapie

Planen Sie, die teilnehmerbezogene Daten anderen Forschern anonymisiert zur Verfügung zu stellen?

Nein

Beschreibung IPD sharing Plan:

[---]*

Organisatorische Daten

- DRKS-ID der Studie: **DRKS00015625**
- Registrierungsdatum im DRKS: **15.10.2019**
- Registrierungsdatum im Partnerregister oder anderem Primärregister: **[---]***
- Wissenschaftsinitiierte Studie (IST/IIT): **ja**
- Antragsdatum bei der (federführenden) Ethikkommission: **30.08.2018**
- Datum des positiven Votums/der zustimmenden Bewertung der (federführenden) Ethikkommission: **12.10.2018**
- Votum der Ethikkommission: **Positives Votum/Zustimmende Bewertung**
- (federführende) Ethikkommissions Vorlage-Nr.: **18-304, Ethik-Kommission der Medizinischen Fakultät der Universität zu Köln**

Sekundäre IDs

- Universal Trial Number (UTN): **U1111-1234-7051**

Untersuchte Krankheit/Gesundheitsproblem

- ICD10: **F91.3 - Störung des Sozialverhaltens mit oppositionellem, aufsässigem Verhalten**
- Freitext: **Dysfunktionale Affektregulation**

Interventionsgruppen/Beobachtungsgruppen

- Arm 1: **Vorgesehen ist eine Gesamtstichprobe von 60 (30/30) Kindern im Alter von sechs bis zwölf Jahren. Diese setzt sich aus jeweils 30 Kindern in der Experimentalgruppe (THAV/ScouT+AUTHARK) und in der Kontrollgruppe (THAV/ScouT) zusammen. Die Rekrutierung der Patienten soll über Zeitungsaufrufe, über das Anfragen örtlicher Schulen, über Spontanmeldungen in der Klinik und beim Ausbildungsinstitut für Kinder- und Jugendlichenpsychotherapie an der Universität Köln (AKiP) erfolgen. Die Rekrutierung beginnt voraussichtlich im Oktober 2019.**
  Die Zuteilung auf die Experimentalgruppe (THAV/ScouT+AUTHARK) (N=30) und die Kontrollgruppe (THAV/ScouT) (N=30) erfolgt randomisiert. Nach Einschluss (T1) durchlaufen die Patient*innen beider Behandlungsgruppen zunächst in Phase 1 und Phase 2 je acht Kind- und zwei Eltern-Sitzungen. Bei der Behandlung handelt es sich um eine ambulante Einzeltherapie. Diese umfasst insgesamt maximal 24 Patient*innen- und 6 Elterntermine. Die Sitzungen finden einmal wöchentlich statt und dauern je 50 Minuten. Die Behandlung wird von den Krankenkassen finanziert.
  Nach jeder Phase (T2, T3) werden von den Patient*innen und den Bezugspersonen mehrere Fragebogen ausgefüllt und Diagnostik zur Verlaufskontrolle durchgeführt. Darüber hinaus wird wöchentlich eine Verhaltensproblemliste zur Verlaufsmessung eingesetzt.
  In der Experimentalgruppe bekommen die Kinder zusätzlich speziell für diesen Zweck aufgesetzte Test-Smartphones, die für die Nutzung der App modifiziert wurden. Diese sollen sie in den verschiedenen Phasen der Therapie einsetzen, um die Diagnostik zu unterstützen (über Momentary-Assessment und Videotagebuch), um Selbstbeobachtungen und Selbstkontrolle zu üben und zu stärken (über das Videotagebuch), um an Therapieaufgaben erinnert zu werden (Erinnerungsfunktion) und um erlernte Inhalte zu trainieren (über die Trainingsfunktion). Die dabei gesammelten Daten enthalten Text- und Videodateien, welche zunächst auf dem Gerät selbst gespeichert werden. Die Daten werden dann von den Therapeut*innen auf einen Computer übertragen und auf nach aktuellen Datenschutzstandards geschützten Laufwerken pseudonymisiert gespeichert. Kinder, die nach 16 Sitzungen klinisch unauffällig sind, erhalten in Phase 3 keine weitere Therapie. Bei ihnen wird nach weiteren acht Wochen eine erste Follow-Up Untersuchung (T4) durchgeführt. Die weiterhin klinisch auffälligen Kinder erhalten weitere acht Sitzungen Therapie, gefolgt von einer abschließenden Untersuchung (T4). Eine weitere Follow-Up Untersuchung (T5) für alle Patient*innen (N=60) findet ein Jahr nach Beendigung

der Therapie statt.

- Arm 2: **Vorgesehen ist eine Gesamtstichprobe von 60 (30/30) Kindern im Alter von sechs bis zwölf Jahren. Diese setzt sich aus jeweils 30 Kindern in der Experimentalgruppe (THAV/ScouT+AUTHARK) und in der Kontrollgruppe (THAV/ScouT) zusammen. Die Rekrutierung der Patienten soll über Zeitungsaufrufe, über das Anfragen örtlicher Schulen, über Spontanmeldungen in der Klinik und beim Ausbildungsinstitut für Kinder- und Jugendlichenpsychotherapie an der Universität Köln (AKiP) erfolgen. Die Rekrutierung beginnt voraussichtlich im Oktober 2019.**
Die Zuteilung auf die Experimentalgruppe (THAV/ScouT+AUTHARK) (N=30) und die Kontrollgruppe (THAV/ScouT) (N=30) erfolgt randomisiert. Nach Einschluss (T1) durchlaufen die Patient*innen beider Behandlungsgruppen zunächst in Phase 1 und Phase 2 je acht Kind- und zwei Eltern-Sitzungen. Bei der Behandlung handelt es sich um eine ambulante Einzeltherapie. Diese umfasst insgesamt maximal 24 Patient*innen- und 6 Elterntermine. Die Sitzungen finden einmal wöchentlich statt und dauern je 50 Minuten. Die Behandlung wird von den Krankenkassen finanziert.
Nach jeder Phase (T2, T3) werden von den Patient*innen und den Bezugspersonen mehrere Fragebogen ausgefüllt und Diagnostik zur Verlaufskontrolle durchgeführt. Darüber hinaus wird wöchentlich eine Verhaltensproblemliste zur Verlaufsmessung eingesetzt.
Kinder der Kontrollgruppe füllen händisch Selbstbeobachtungsbogen aus, die dem Schema des Videotagebuchs der Experimentalgruppe entsprechen.
Kinder, die nach 16 Sitzungen klinisch unauffällig sind, erhalten in Phase 3 keine weitere Therapie. Bei ihnen wird nach weiteren acht Wochen eine erste Follow-Up Untersuchung (T4) durchgeführt. Die weiterhin klinisch auffälligen Kinder erhalten weitere acht Sitzungen Therapie, gefolgt von einer abschließenden Untersuchung (T4). Eine weitere Follow-Up Untersuchung (T5) für alle Patient*innen (N=60) findet ein Jahr nach Beendigung der Therapie statt.

Charakteristika

- Studientyp: **Interventionell**
- Studientyp nicht-interventionell: **[---]***
- Studiendesign Zuteilung: **Kontrollierte, randomisierte Studie**
- Verblindung: **Offen**
- Wer ist verblindet: **[---]***
- Kontrolle: **Aktive Kontrolle (wirksame Behandlung der Kontrollgruppe)**
- Studienzweck: **Therapie**
- Gruppenzuteilung: **Parallelverteilung**
- Studienphase: **Nicht zutreffend**
- Off-label use (Zulassungsüberschreitende Anwendung eines Arzneimittels): **Nicht zutreffend**

Primärer Endpunkt

In der Experimentalgruppe THAV/ScouT+AUTHARK zeigt sich eine signifikant stärkere Verbesserung der Compliance als in der Kontrollgruppe THAV/ScouT. Compliance meint die Umsetzung der Therapieaufgaben im Alltag. Die Compliance wird über einen Fragebogen (FB Compliance) erhoben. Mit dem Fragebogen zur Behandlungs-Compliance können die Therapeut*innen im Anschluss an jede Therapiesitzung die Mitarbeit des Kindes und der Eltern, sowie die Nutzung der App beurteilen. Für die Studie wurde der Compliance Fragebogen nach Görtz-Dorten & Döpfner (2008) modifiziert. Eine empirische Prüfung der ursprünglichen Version des Fragebogens ergab interne Konsistenzen zwischen $\alpha \geq .948$ bzw. $\alpha \geq .954$ bei den Kindern und $\alpha \geq .968$ bzw. $a \geq .965$ bei den Eltern (Faber, 2014; Stadler, 2018). Die modifizierte Version umfasst 25 Items, welche um die Nutzungserfassung der einzelnen App-Funktionen bzw. die Erledigung der täglichen Selbstbeobachtungen und die Bewertung der Qualität der Einträge erweitert wurde.

Sekundärer Endpunkt

In der Therapiegruppe THAV/ScouT+AUTHARK zeigt sich eine signifikant stärkere Reduktion der aggressiven Symptomatik als in der Therapiegruppe THAV/ScouT. Die aggressive Symptomatik wird über Fremdbeurteilungs- und Selbstbeurteilungsbögen zu den Messzeitpunkten 1 bis 5 erfasst: Fremdbeurteilungsbogen für Störung des Sozialverhaltens (FBB- SSV, aus DISYPS III, Döpfner & Görtz-Dorten, 2016).

Selbstbeurteilungsbogen für Störung des Sozialverhaltens (SBB- SSV aus DISYPS III, Döpfner & Görtz-Dorten, 2016).
Fragebogen zum aggressiven Verhalten (Fremdurteil) (FAVK- F, Görtz-Dorten & Döpfner, 2010)
Fragebogen zum aggressiven Verhalten (Fremdurteil) (FAVK- S, Görtz-Dorten & Döpfner, 2010)
Die aggressive Symptomatik wird außerdem über eine wöchentliche Problemliste aus dem THAV Manual, sowie das tägliche Momentary Assessment und das Videotagebuch in der Smartphone App AUTHARK erfasst.
Die komorbide Symptomatik wird über folgende Instrumente erfasst:
Fremdbeurteilungsbogen für Aufmerksamkeitsdefizit-/ Hyperaktivitätsstörung (FBB-ADHS; Döpfner & Görtz-Dorten, 2016).
Selbstbeobachtungsbogen für Aufmerksamkeitsdefizit-/ Hyperaktivitätsstörung (SBB-ADHS; Döpfner & Görtz-Dorten, 2016)
Elternfragebogen über das Verhalten von Kindern und Jugendlichen (CBCL/6-18R; Döpfner, Plück, Kinnen, & Arbeitsgruppe Deutsche Child Behavior Checklist, 2014)
Lehrerfragebogen über das Verhalten von Kindern und Jugendlichen (TRF 6-19R; Döpfner, Plück, Kinnen, & Arbeitsgruppe Deutsche Child Behavior Checklist, 2014)

Die psychische und die psychosoziale Funktion, die Lebensqualität der Patienten, die Familienbelastungen sowie Psychopathologie der Eltern wird mit folgenden Instrumenten erfasst:
Test zur Erfassung sozial-kognitiver Informationsverarbeitung (ScouT-Diagnostik; Görtz-Dorten & Döpfner, 2016)
Fragebogen zum psychosozialen Funktionsniveau (Goertz-Dorten et al., 2018)
Fragebogen zur Lebensqualität (KINDL-R; Ravens-Sieberer & Bullinger, 2003)
Depressions-Angst-Stress-Fragebogen (DASS; Essau, 1995)
Fragebogen zur elterlichen Aggression (FB-Ä: modifiziert nach Bryant & Smith, 2001)
Fragebogen zur Empathie „Callous-Unemotional Traits“ (ICU; Essau, Sasagawa & Frick, 2006; Frick, 2003)
Fragebogen zur Regulation unangenehmer Stimmungen von Kindern (FRUST; basierend auf Feel-KJ; Grob & Smolenski, 2009)

Die Therapeuten-Patienten-Beziehung, die Behandlungszufriedenheit sowie die Behandlungsintegrität werden mit folgenden Instrumenten beurteilt:
Beziehungsfragebogen für die Kinder- und Jugendlichenpsychotherapie (BeKi; Kinnen, Breuer & Döpfner, 2011)
Fragebogen zur Behandlungszufriedenheit, Client Satisfaction Questionnaire (CSQ-8; Attkisson, 2012)
Fragebogen zur Behandlungs-Integrität (Görtz-Dorten)

Länder in denen Studienteilnehmer rekrutiert werden

- DE: **Deutschland**

Rekrutierungsstandorte

- **Klinik** Ambulanz des Ausbildungsinstituts für Kinder- und Jugendpsychotherapie der Uniklinik Köln, Köln

Rekrutierung

- Geplant/Tatsächlich: **Tatsächlich**
- (geplantes/tatsächliches Datum) Einschluss des ersten Studienteilnehmers: **01.11.2019**
- Geplante Studienteilnehmeranzahl gesamt: **60**
- Monozentrisch/Multizentrisch: **Monozentrisch**
- National/International: **National**

Einschlusskriterien

- Geschlecht: **Beide, männlich und weiblich**
- Mindestalter: **72 Monate**
- Höchstalter: **143 Monate**

Weitere Einschlusskriterien

Einschlusskriterien sind die klinische Diagnose einer Störung des Sozialverhaltens mit oppositionellem, aufsässigem Verhalten (ICD-10: F91.3) und die Erfüllung des Items „häufiges Beginnen von körperlichen Auseinandersetzungen (außer Geschwisterauseinandersetzungen)" im klinischen Urteil. Um die Diagnosekriterien zu überprüfen, wird die Diagnosecheckliste für Störungen des Sozialverhaltens (DCL-SSV) aus dem DISYPS III (Döpfner et al. 2016) eingesetzt. Außerdem wird mit Hilfe des FBB-SSV aus dem DISYPS-III (Döpfner et al. 2016) ein Score erhoben, der im Fremdurteil (Eltern) bei einem Stanine-Wert von größer/gleich 7 zum Messzeitpunkt 1 (Patient*inneneinschluss) liegen muss.

Ausschlusskriterien

Ausschlusskriterien ist die Diagnose einer Tiefgreifenden Entwicklungsstörung (ICD-10: F84.-) oder einer anderen psychischen Störungen, die im Vordergrund steht, ein IQ unter 80 (CFT 1-R, CFT 20-R) und krisenhafte Zuspitzungen, die eine stationäre Behandlung nahelegen. Ferner soll die Indikation für den Neubeginn einer Pharmakotherapie bzw. für eine Dosierungsveränderung einer bestehenden Pharmakotherapie zum Zeitpunkt der Intervention ausgeschlossen werden. Ebenso sollte das Kind keine andere Psychotherapie erhalten und die Eltern oder Bezugspersonen sollten über ausreichend Deutschkenntnisse verfügen.

Adressen

Primärer Sponsor

Ausbildungsinstitut für Kinder- und Jugendlichenpsychotherapie der Uniklinik Köln (Akip)
Pohligstr. 9
50969 Köln
Deutschland
Telefon: **[---]***
Fax: **[---]***
E-Mail: **[---]***
URL der Einrichtung: http://akip.uk-koeln.de/

Kontakt für wissenschaftliche Anfragen

Ausbildungsinstitut für Kinder- und Jugendlichenpsychotherapie der Uniklinik Köln Evaluation/ Forschungsbereich Psychotherapie; Kölner Institut der Christoph-Dornier-Stiftung fürKlinische Kinderpsychologie
Frau Priv. Doz. Dr. rer medic. Anja Görtz-Dorten
Pohligstr. 9
50969 Köln
Deutschland
Telefon: **0221 478-76836**
Fax: **[---]***
E-Mail: **anja.goertz-dorten at uk-koeln.de**
URL der Einrichtung: [---]*

Kontakt für Studienteilnehmer

Ausbildungsinstitut für Kinder- und Jugendlichenpsychotherapie der Uniklinik Köln Evaluation/ Forschungsbereich Psychotherapie; Kölner Institut der Christoph-Dornier-Stiftung fürKlinische Kinderpsychologie
Frau Priv. Doz. Dr. rer medic. Anja Görtz-Dorten
Pohligstr. 9
50969 Köln
Deutschland
Telefon: **0221 478-76836**
Fax: **[---]***
E-Mail: **anja.goertz-dorten at uk-koeln.de**
URL der Einrichtung: [---]*

Finanzierungsquellen

Haushaltsmittel, keine fremden Finanzmittel (Budget des Studienleiters)

Ausbildungsinstitut für Kinder- und Jugendlichenpsychotherapie der Uniklinik Köln (Akip)
Pohligstr. 9
50969 Köln
Deutschland
Telefon: **[---]***
Fax: **[---]***
E-Mail: **[---]***
URL der Einrichtung: http://akip.uk-koeln.de/

Private Gelder (Stiftungen, Studiengesellschaften etc.)
Christiph Dornier Stiftung für klinische Psychologie Institut Köln
Pohligstr. 9
50969 Köln
Deutschland
Telefon: **[---]***
Fax: **[---]***
E-Mail: **[---]***
URL der Einrichtung: https://www.christoph-dornier-stiftung.de/institut_koeln.html

Status

- Status der Rekrutierung: **Rekrutierung läuft**
- Grund, falls "Status der Rekrutierung" "permanent eingestellt" oder "zurückgezogen": **[---]***
- Grund, falls Grund des Rekrutierungsstopps "Anderer": **[---]***
- Tatsächliches Datum des Studienabschlusses (LPLV): **[---]***
- Tatsächliche Gesamtzahl Studienteilnehmer in Deutschland nach abgeschlossener Rekrutierung: **[---]***
- Tatsächliche Gesamtzahl Studienteilnehmer aller Zentren nach abgeschlossener Rekrutierung: **[---]***

Publikationen, Studienergebnisse und weitere Studiendokumente

- **[---]***

Alle grau hinterlegten Felder werden in der öffentlichen Ansicht nicht angezeigt.
** Dieser Eintrag bedeutet, dass der Parameter entweder nicht zutrifft oder dass er nicht eingetragen wurde.*

Vorschau der Studiendaten gedruckt am 09. June 2021 12:40:38

DRKS-ID: **DRKS00015625**

Trial Description

Title

Evaluation of a smartphone-app-supported intervention (AUTHARK) combined with the multimodal treatment program for children with aggressive behaviour (THAV) and the social computer based competence training for children with aggressive behaviour (ScouT) in comparison to the treatment programs THAV and ScouT without smartphone-app support.

Trial Acronym

AUTHARK

URL of the Trial

http://uk-koeln.de/authark

Brief Summary in Lay Language

The treatment program for children with aggressive behaviour (THAV; Görtz-Dorten & Döpfner, 2019) and the social computer based competence training for children with aggressive behaviour (ScouT; Görtz-

Dorten & Döpfner, 2016) were developed for children ages 6 to 12 at the School for Child and Adolescent Psychotherapy at the University of Cologne. Both programmes focus on training of social information processing, developing and strengthening impulse control, social competence training and modifying social interactions. Preliminary studies have already demonstrated that the programmes are highly effective.
The present study examines the additional effects of a therapy-supporting smartphone app (AUTHARK; Görtz-Dorten & Döpfner; 2019) in children treated with THAV and ScouT. The treatment supporting smartphone app is based on THAV and ScouT. It contains functions that facilitate the transfer of therapy contents into everyday life, but also make everyday situations more accessible for therapy. These functions include a video diary, a mood and behaviour momentary assessment, a therapy task reminder function, and a training function for the therapy contents.
We intend to investigate whether the combined treatment with the smartphone app increases homework compliance and leads to a better implementation of the therapy tasks in everyday life. Furthermore, the study should show whether the additional use of the app during therapy reduces the aggressive or other problematic behaviour more than a therapy without the supporting app.
We recruit a sample of 60 children between the ages of 6 and 12 with aggressive behaviour towards peers. First, all children undergo a detailed initial examination via questionnaires and personal interviews with children, caregivers and teachers. Then we randomly assign the children to one of the two treatment groups.
One half of the children (50%) is treated with the THAV & ScouT therapy programmes. The second half of the children (50%) is treated with the therapy programs THAV & ScouT and the treatment-supporting smartphone app AUTHARK.
Therapy sessions take place weekly. During the treatment, observation sheets or video diaries are kept every two weeks. In addition, further questionnaires are completed at three fixed assessment points. These questionnaires will be used to check the implementation of the therapy tasks, the course of the problems during the therapy and the effectiveness of the treatment.
The group additionally treated with the AUTHARK app, receives a smartphone for the treatment period. The therapy within the study comprises 16 to 24 patient contacts, plus a maximum of six sessions with significant others. As a rule, this means that the total duration of participation is at least 24 weeks. Participation is open to families whose children are between 6 and 12 years of age and show aggressive behaviour towards their peers. In addition, conditions for conducting the study must be fulfilled (e.g. sufficient knowledge of the German language among children and parents).

Brief Summary in Scientific Language

At the School for Child and Adolescent Psychotherapy at the University of Cologne, two therapy programmes for children aged six to twelve with aggressive behavioural disorders were developed: THAV and ScouT.
THAV and ScouT are multimodal treatment programmes that combine patient-centred and environment-centred interventions and thus comply with the national guidelines for the therapy of children with aggressive behaviour. These two therapy programs use cognitive and behavioral treatment techniques that have been proven to be very effective in research and in practice. Patient-centered intervention uses situations in which the children show aggressive behaviour towards their peers. The focus here is on the training of social cognitive information processing, the development and strengthening of impulse control, social competence training and the modification of social interactions. In addition, the therapy programmes include family and school-centred interventions. Several studies show the effectiveness of THAV and ScouT (Goertz-Dorten et al. 2017, Goertz-Dorten et al. 2018a; Goertz-Dorten et al., 2018b; Goertz-Dorten et al., accepted; Lindenschmidt, 2016).
The aim of this study is to evaluate a specifically developed therapy smartphone app for children with aggressive behaviour (AUTHARK).
The smartphone app, which is based on the THAV and ScouT therapy programs, contains the following functions: a mood and behaviour momentary assessment, a video diary function, a therapy task reminder function and a training function. Thus, the smartphone records specific information about conflict situations and emotions children experience in everyday life, and supports the performance of therapy tasks. Thereby, the transfer of coping strategies, developed in therapy, into the everyday life of the children can be supported and checked. Furthermore, the motivation to carry out the therapy tasks increases.

The aim of the overall project is to compare the effects of a treatment with the THAV and SCOUT therapy programs combined with AUTHARK and a treatment with the two therapy programs without additional app support.
For this purpose, we developed the following hypotheses, which will be tested in randomized control group design.
1.) The experimental group THAV/ScouT + AUTHARK shows a stronger improvement in compliance (implementation of therapy tasks) than the control group THAV/ScouT (recorded via FB Compliance, after each session). (primary outcome)
2.) The experimental group THAV/ScouT+AUTHARK shows a stronger reduction of aggressive symptoms than the control group THAV/ScouT (assessed via FBB-SSV parents, FBB-SSV teachers, SBB-SSV, individual problem list).
3.) The experimental group THAV/ScouT+AUTHARK shows a stronger reduction of comorbid symptoms than the control group THAV/ScouT (assessed by CBCL-Total, TRF-Total, YSR-Total, FBB-ADHS parents, FBB-ADHS teachers, SBB-ADHS, DADYS-E/K and FRUST-F/S).
4.) The experimental group THAV/ScouT + AUTHARK shows a stronger improvement of the patients psychological functions that contribute to the development of aggressive symptoms (social-cognitive information processing, impulse control, social skills, empathy) than the control group THAV/ScouT (assessed via FAVK-F/FAVK-S, ICU-E/K, ScouT diagnostics).
5.) The experimental group THAV/ScouT + AUTHARK a stronger improvement of the patients psychosocial functional level and the quality of life is shown than in the control group ScouT/THAV (assessed via the scale to the functional level, KINDL-R).
6.) The experimental group ScouT/THAV+AUTHARK shows a higher satisfaction of patients, parents and therapists than the control group ScouT/THAV (assessed via CSQ).

Keywords

Behavioural therapy for children, Aggressive behaviour, Evaluation study, Smartphone-supported behavioural therapy

Do you plan to share individual participant data with other researchers?

No

Description IPD sharing plan:

[---]*

Organizational Data

- DRKS-ID: **DRKS00015625**
- Date of Registration in DRKS: **2019/10/15**
- Date of Registration in Partner Registry or other Primary Registry: **[---]***
- Investigator Sponsored/Initiated Trial (IST/IIT): **yes**
- Date of (leading) Ethics Committee Application: **2018/08/30**
- Date of (leading) Ethics Committee Approval: **2018/10/12**
- Ethics Approval/Approval of the Ethics Committee: **Approved**
- (leading) Ethics Committee No.: **18-304, Ethik-Kommission der Medizinischen Fakultät der Universität zu Köln**

Secondary IDs

- Universal Trial Number (UTN): **U1111-1234-7051**

Health Condition or Problem studied

- ICD10: **F91.3 - Oppositional defiant disorder**
- Free text: **Affective Dysregulation**

Interventions/Observational Groups

- Arm 1: **A total sample of 60 (30/30) children aged six to twelve years is planned. It consists of 30 children each in the experimental group (THAV/ScouT+AUTHARK) and in the control group (THAV/ScouT). Recruitment of the patients is to take place via newspaper calls, via enquiries from local schools, via spontaneous registrations at the outpatient untis of the Clinic for Child and Adolescent Psychiatry and at the School for Child and Adolescent Psychotherapy (AKiP) at the University of Cologne. Recruitment is expected to begin in october 2019.**
  The allocation to the experimental group (THAV/ScouT+AUTHARK) (N=30) and the control group (THAV/ScouT) (N=30) will be random. After the inclusion of all patients (T1), both treatment groups initially undergo eight child and two parent sessions each in phase 1 and phase 2. The treatment is an outpatient individual therapy. This comprises a maximum of 24 patient sessions and 6 sessions with the parents. The sessions take place once a week and last 50 minutes each. Health insurance companies cover the treatment.
  After each phase (T2, T3), we collect several patient-, caregiver- and teacher-ratings and perform diagnostic investigations to monitor the progress. In addition, a weekly behavioural problem list measures the course of the disorder.
  In the experimental group, the children receive additionally test smartphones specially set up for the study, modified for use with the smartphone app only. These are used in the various phases of therapy to support diagnosis (via momentary assessment and video diary), to practice and strengthen self-observation and self-control (via video diary), to remind of therapy tasks (reminder function) and to train learned therapy content (training function). The data collected, contains text and video files that are first stored on the device itself. Then the therapist transfers the data to a computer and stores it pseudonymously on drives protected according to current data protection standards. Children who are below the clinical cut off for aggressive behavior after 16 sessions do not receive any further therapy in phase 3. A first follow-up examination (T4) is performed after a further eight weeks. Children who remain above the clinical cut off receive a further eight sessions of therapy, followed by a final examination (T4). Another follow-up examination (T5) for all patients (N=60) takes place one year after the end of therapy.
- Arm 2: **A total sample of 60 (30/30) children aged six to twelve years is planned. It consists of 30 children each in the experimental group (THAV/ScouT+AUTHARK) and in the control group (THAV/ScouT). Recruitment of the patients is to take place via newspaper calls, via enquiries from local schools, via spontaneous registrations at the outpatient untis of the Clinic for Child and Adolescent Psychiatry and at the School for Child and Adolescent Psychotherapy (AKiP) at the University of Cologne. Recruitment is expected to begin in october 2019.**
  The allocation to the experimental group (THAV/ScouT+AUTHARK) (N=30) and the control group (THAV/ScouT) (N=30) will be random. After the inclusion of all patients (T1), both treatment groups initially undergo eight child and two parent sessions each in phase 1 and phase 2. The treatment is an outpatient individual therapy. This comprises a maximum of 24 patient sessions and 6 sessions with the parents. The sessions take place once a week and last 50 minutes each. Health insurance companies cover the treatment.
  After each phase (T2, T3), we collect several patient-, caregiver- and teacher-ratings and perform diagnostic investigations to monitor the progress. In addition, a weekly behavioural problem list measures the course of the disorder.
  Children in the control group manually fill out self-observation forms that correspond to the scheme of the video diary in the experimental group.
  Children who are below the clinical cut off for aggressive behavior after 16 sessions do not receive any further therapy in phase 3. A first follow-up examination (T4) is performed after a further eight weeks. Children who remain above the clinical cut off receive a further eight sessions of therapy, followed by a final examination (T4). Another follow-up examination (T5) for all patients (N=60) takes place one year after the end of therapy.

Characteristics

- Study Type: **Interventional**
- Study Type Non-Interventional: **[---]***
- Allocation: **Randomized controlled trial**
- Blinding: **Open (masking not used)**
- Who is blinded: **[---]***

- Control: **Active control (effective treament of control group)**
- Purpose: **Treatment**
- Assignment: **Parallel**
- Phase: **N/A**
- Off-label Drug use: **N/A**

Primary Outcome

The experimental group THAV/ScouT+AUTHARK will show a significantly stronger improvement in compliance than the control group THAV/ScouT. Compliance means the implementation of therapy tasks in everyday life. A questionnaire assesses the compliance (FB Compliance). With the questionnaire on treatment compliance, the therapist can assess the cooperation of the child and the parents as well as the use of the app or homework compliance after each therapy session. For the study, the compliance questionnaire according to Görtz-Dorten & Döpfner (2008) was modified. An empirical analysis of the original questionnaire version showed internal consistencies between α ≥ .948 or α ≥ .954 for children and α≥ .968 or a ≥ .965 for parents (Faber, 2014; Stadler, 2018). The modified version contains 25 items, which were extended by the usage recording of the individual app functions or the completion of the daily self-observations and the evaluation of the entry quality.

Secondary Outcome

**The therapy group THAV/ScouT+AUTHARK shows a significantly stronger decrease of aggressive symptoms than the therapy group THAV/ScouT. The aggressive symptoms are assessed using external and self-assessment questionnaires at measurement points 1 to 5:
Parent- and teacher rated aggressive behaviour, CD and ODD (FBB- SSV, from DISYPS III, Döpfner & Görtz-Dorten, 2016).
Self-rated rated aggressive behaviour, CD and ODD (SBB- SSV from DISYPS III, Döpfner & Görtz-Dorten, 2016).
Questionnaire on aggressive behaviour (external assessment) (FAVK-F, Görtz-Dorten & Döpfner, 2010)
Questionnaire on aggressive behaviour (Self- assessment) (FAVK-F, Görtz-Dorten & Döpfner, 2010)
The aggressive symptoms are additionally assessed via a weekly problem list from the THAV manual, as well as the daily Momentary Assessment and the video diary in the Smartphone App AUTHARK at the various assessment points.
Comorbid symptoms are assessed using the following instruments:
Questionnaire for attention deficit hyperactivity disorder (external assessment) (FBB-ADHS; Döpfner & Görtz-Dorten, 2016).
Questionnaire for Attention Deficit Hyperactivity Disorder (Self-assessment) (SBB-ADHS; Döpfner & Görtz-Dorten, 2016)
Parent questionnaire on the behaviour of children and adolescents (CBCL/6-18R; Döpfner, Plück, Kinnen, & Arbeitsgruppe Deutsche Child Behavior Checklist, 2014)
Teacher Questionnaire on Child and Adolescent Behavior (TRF 6-19R; Döpfner, Plück, Kinnen, & Arbeitsgruppe Deutsche Child Behavior Checklist, 2014)**

**The psychological and psychosocial functions, the quality of life of the patients, the family impairments as well as the psychopathology of the parents are assessed with the following instruments:
Test for recording social-cognitive information processing (ScouT-Diagnostics; Görtz-Dorten & Döpfner, 2016)
Questionnaire on psychosocial functional level (Goertz-Dorten et al., 2018)
Questionnaire on quality of life (KINDL-R; Ravens-Sieberer & Bullinger, 2003)
Depression Anxiety Stress Questionnaire (DASS; Essau, 1995)
Questionnaire on parental aggression (FB-Ä: modified after Bryant & Smith, 2001)
Questionnaire on Empathy "Callous-Unemotional Traits" (ICU; Essau, Sasagawa & Frick, 2006; Frick, 2003)
Questionnaire on the Regulation of Dissatisfactory Moods in Children (FRUST; based on Feel-KJ; Grob & Smolenski, 2009)**

The therapist-patient relationship, treatment satisfaction and treatment integrity are assessed using the following instruments:

Questionnaire on the patient-therapist relationship in Child and Adolescent Psychotherapy (BeKi; Kinnen, Breuer & Döpfner, 2011)
Client Satisfaction Questionnaire (CSQ-8; Attkisson, 2012)
Questionnaire on treatment integrity (Görtz-Dorten)

Countries of Recruitment

- DE: **Germany**

Locations of Recruitment

- **Medical Center** Ambulanz des Ausbildungsinstituts für Kinder- und Jugendpsychotherapie der Uniklinik Köln, Köln

Recruitment

- Planned/Actual: **Actual**
- (Anticipated or Actual) Date of First Enrollment: **2019/11/01**
- Target Sample Size: **60**
- Monocenter/Multicenter trial: **Monocenter trial**
- National/International: **National**

Inclusion Criteria

- Gender: **Both, male and female**
- Minimum Age: **72 Months**
- Maximum Age: **143 Months**

Additional Inclusion Criteria

One of the inclusion criteria is the clinical diagnosis of ODD (ICD-10: F91.3). Furthermore, children had to show peer-related aggressive behavior causing persistent impairment of relationships with other children (clinical rating on the basis of a semi-structured interview) and a high total score (Stanine ≥ 7) in parent rating on the Symptom Checklist for Disruptive Behavior Disorder (SCL-DBD) of the DISYPS-III (Döpfner et al., 2016).

Exclusion Criteria

Exclusion criteria are the presence of a primary comorbid disorder (e.g., autism) according to the judgment of the clinician, an IQ score below 80 (CFT 1-R, CFT 20-R) or crises that indicate inpatient treatment, a planned change in medication in a child receiving psychotropic medication, other child psychotherapy, and parents or caregivers who do not speak sufficient German.

Addresses

Primary Sponsor

Ausbildungsinstitut für Kinder- und Jugendlichenpsychotherapie der Uniklinik Köln (Akip)
Pohligstr. 9
50969 Köln
Germany
Telephone: **[---]***
Fax: **[---]***
E-mail: **[---]***
URL: http://akip.uk-koeln.de/

Contact for Scientific Queries

Ausbildungsinstitut für Kinder- und Jugendlichenpsychotherapie der Uniklinik Köln Evaluation/ Forschungsbereich Psychotherapie; Kölner Institut der Christoph-Dornier-Stiftung fürKlinische Kinderpsychologie
Ms. Priv. Doz. Dr. rer medic. Anja Görtz-Dorten
Pohligstr. 9
50969 Köln

Germany
Telephone: **0221 478-76836**
Fax: **[---]***
E-mail: **anja.goertz-dorten at uk-koeln.de**
URL: [---]*

Contact for Public Queries

Ausbildungsinstitut für Kinder- und Jugendlichenpsychotherapie der Uniklinik Köln Evaluation/ Forschungsbereich Psychotherapie; Kölner Institut der Christoph-Dornier-Stiftung fürKlinische Kinderpsychologie
Ms. Priv. Doz. Dr. rer medic. Anja Görtz-Dorten
Pohligstr. 9
50969 Köln
Germany
Telephone: **0221 478-76836**
Fax: **[---]***
E-mail: **anja.goertz-dorten at uk-koeln.de**
URL: [---]*

Sources of Monetary or Material Support

Institutional budget, no external funding (budget of sponsor/PI)

Ausbildungsinstitut für Kinder- und Jugendlichenpsychotherapie der Uniklinik Köln (Akip)
Pohligstr. 9
50969 Köln
Germany
Telephone: **[---]***
Fax: **[---]***
E-mail: **[---]***
URL: http://akip.uk-koeln.de/

Private sponsorship (foundations, study societies, etc.)

Christiph Dornier Stiftung für klinische Psychologie Institut Köln
Pohligstr. 9
50969 Köln
Germany
Telephone: **[---]***
Fax: **[---]***
E-mail: **[---]***
URL: https://www.christoph-dornier-stiftung.de/institut_koeln.html

Status

- Recruitment Status: **Recruiting ongoing**
- Reason, if "Recruitment stopped after recruiting started" or "Recruiting withdrawn before recruting started": **[---]***
- Reason, if Reason for Recruiting Stop "Other": **[---]***
- Study Closing (LPLV): **[---]***
- Number of Participants in Germany after Recruiting complete: **[---]***
- Total Number of Participants (all Sites worldwide) after Recruiting complete: **[---]***

Trial Publications, Results and other Documents

- **[---]***

All grey highlighted fields will not be displayed on the public web site.
** This entry means the parameter is not applicable or has not been set.*
