# Supplementary material for: Effects of a smartphone app-augmented treatment for children with oppositional defiant disorder / conduct disorder and peer-related aggressive behavior – a pilot study
Source: Trials. 2022 Jul 8;23:554. doi: 10.1186/s13063-022-06325-6 (PMC9264298; doi:10.1186/s13063-022-06325-6)
Supplement: Supplementary file 2 — Additional file 2: Model written informed consent. [file 13063_2022_6325_MOESM2_ESM.pdf]

UNIKLINIK KÖLN

Ausbildungsinstitut für Kinder- und Jugendlichenpsychotherapie (AKiP) an der Klinik für Psychiatrie, Psychosomatik und Psychotherapie des Kindes- und Jugendalters

akipköln - kjpppköln

AUTHARK

APP-UNTERSTÜTZTE THERAPIE-ARBEIT FÜR KINDER

Forschungsprojekt AUTHARK

(„**A**pp-**u**nterstützte **Th**erapie-***Ar****beit für* ***K****inder“)*

Evaluation der Wirksamkeit einer app-unterstützten Therapie (AUTHARK) in Kombination mit dem multimodalen Therapieprogramm zur Behandlung von Kindern mit aggressivem Verhalten (THAV) und des Sozialen computerunterstützten Trainings (ScouT) im Vergleich zu einer nicht app-unterstützten Behandlung mit den beiden Programmen.

Hilfen für Kinder mit aggressivem Verhalten und affektiver Dysregulation

- Informationen über die Studie -

Liebe Eltern,

wir möchten Sie fragen, ob Sie und Ihr Kind bereit dazu sind, an unserer Studie teilzunehmen. Bevor Sie und Ihr Kind sich damit einverstanden erklären, sollten Sie Ziel und Inhalt dieser Studie verstehen. Die vorliegende Einwilligungserklärung erläutert, warum wir diese Studie durchführen. Bitte lesen Sie den folgenden Text als Ergänzung zum Informationsgespräch sorgfältig durch und zögern Sie nicht, Fragen zu stellen.

1. Warum führen wir diese Studie durch?

Aggressives Verhalten ist einer der häufigsten Vorstellungsgründe in der Kinder- und Jugendlichenpsychotherapie. In unserem Haus wurden Therapieprogramme für Kinder mit dieser Symptomatik entwickelt. Dabei handelt es sich zum einen um das Therapieprogramm für Kinder mit aggressivem Verhalten (THAV) und zum anderen um das Soziale computergestützte Training für Kinder mit aggressivem Verhalten (ScouT). In einer Untersuchung soll die Wirksamkeit der Programme in Kombination mit einer therapieunterstützenden App (AUTHARK) überprüft werden. Es gibt bereits Voruntersuchungen, die den Programmen eine hohe Wirksamkeit bescheinigen.

Allgemeines zu den Therapieprogrammen THAV & ScouT

Bei den Therapieprogrammen kommen Behandlungsmethoden zum Einsatz, die sich bereits als sehr wirksam erwiesen und die sich in der Klinik sehr bewährt haben. Die Schwerpunkte der Programme sind u.a.:

- Soziales Kompetenztraining
- Ärgerkontrolltraining
- Stärkung von Impulskontrolle
- Familien- und schulzentrierte Interventionen

Allgemeines zur Therapie-App AUTHARK

Die therapieunterstützende App baut auf den Therapieprogrammen THAV und ScouT auf. Sie enthält Funktionen, die den Übertrag von Therapieinhalten in den Alltag erleichtern sollen, aber auch Alltagssituationen für die Therapie leichter zugänglich machen. Zu diesen Funktionen zählen das Führen eines Videotagebuchs, eine Momentaufnahme-Funktion, bei der zu definierten Zeitpunkten die aktuellen Gefühle in Verbindung mit aktuellem Verhalten abgefragt werden, die Erinnerung an individuell vereinbarte Therapieaufgaben, sowie eine Trainingsfunktion der erarbeiteten Inhalte.

Die Therapieprogramme bieten kindgerechte Arbeitsmaterialien für 6 bis 12-jährige Kinder.

Ziel der Untersuchung ist es, die Wirksamkeit der Therapie in Kombination mit den neuen Arbeitsmaterialien nachzuweisen. Dazu ist es notwendig, Informationen über die Entwicklung des aggressiven Verhaltens Ihres Kindes gegenüber Gleichaltrigen zu erhalten. Viele dieser Informationen werden in Form von Fragebögen schriftlich erfasst. Wir bemühen uns, diesen Aufwand so gering wie möglich zu halten. Alle Informationen, die Sie uns mitteilen, unterliegen der Schweigepflicht. Für die Auswertung werden die persönlichen Daten pseudonymisiert.

2. Wie ist der Ablauf der Studie?

An der Studie nehmen insgesamt 60 Kinder zwischen 6 und 12 Jahren mit aggressivem Verhalten gegenüber Gleichaltrigen teil. Zunächst durchlaufen alle Kinder eine ausführliche Eingangsuntersuchung. Hierzu wird eine Reihe von Fragebogen verteilt und persönliche Interviews geführt. Im Gegensatz zu einer Behandlung außerhalb der Studie sind die Befragungen ausführlicher und es werden mehr Fragebogen verteilt.

Die Wirksamkeit der Therapieprogramme in Kombination mit der Smartphone App wird im direkten Vergleich mit einer Behandlung, in der die beiden Programme ohne die zusätzliche App eingesetzt werden, erfasst. Um andere Einflüsse auf die Wirksamkeit so gering wie möglich zu halten, werden die Kinder nach dem Zufallsprinzip in zwei gleich große Gruppen aufgeteilt.

Die eine Hälfte der Kinder (50%) wird mit den Therapieprogrammen THAV & ScouT behandelt.

Die zweite Hälfte der Kinder (50%) wird mit den Therapieprogrammen THAV & ScouT, sowie der therapieunterstützenden App AUTHARK behandelt.

Es werden wöchentliche Therapiestunden stattfinden. Während der Therapie bitten wir Sie, wöchentliche Beobachtungsbögen zu führen und zusätzlich zu drei weiteren Messzeitpunkten einige Fragebögen auszufüllen, auch hier ist der Umfang im Vergleich zu einer Behandlung außerhalb der Studie etwas größer. Anhand dieser Bögen können wir dann den Verlauf der Probleme während der Therapie und die Wirksamkeit der Behandlung überprüfen.

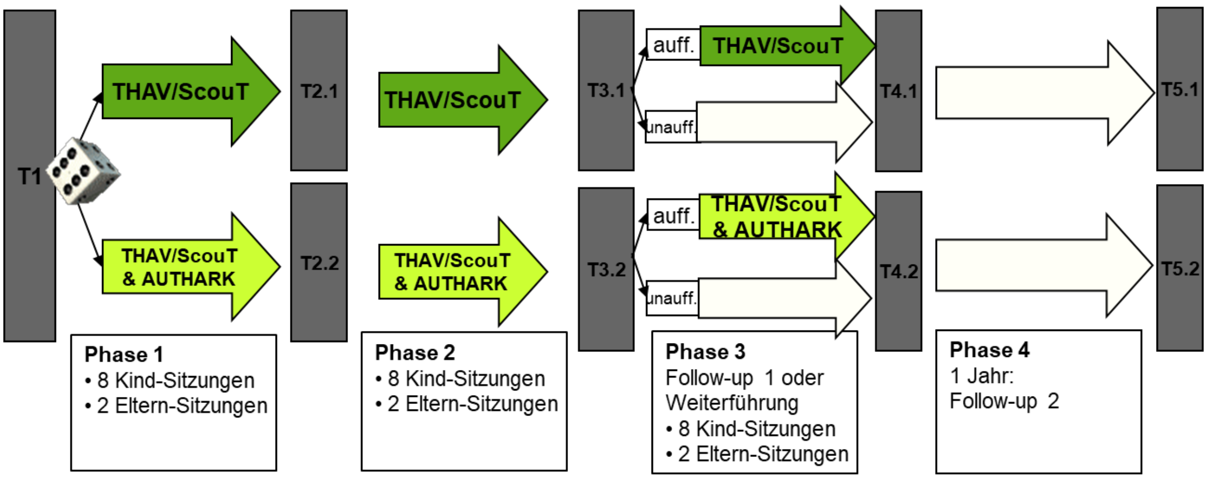

Die Gruppe, die zusätzlich mit der App AUTHARK behandelt wird, bekommt über einen gemeinsam definierten Zeitraum ein Smartphone zur Verfügung gestellt. Dieses ist ausschließlich zur Verwendung der App eingestellt, alle anderen Funktionen sind gesperrt. Hier sollen je

nach Studienphase z.B. täglich Videotagebücher geführt werden oder mehrmals täglich eine Momentaufnahme ausgefüllt werden.

Wenn die angebotenen Behandlungsmaßnahmen nicht den gewünschten Erfolg zeigen, werden wir nach Abschluss der Untersuchung mit Ihnen überlegen, welche anderen Hilfen wir oder andere Einrichtungen anbieten können.

Falls im Laufe der Behandlung deutlich wird, dass alternative oder intensivere therapeutische Maßnahmen notwendig sind, kann die Teilnahme an der Studie jederzeit beendet werden.

3. Wer darf an der Studie teilnehmen?

Teilnehmen können Familien, deren Kinder zwischen 6 und 12 Jahren sind und aggressives Verhalten gegenüber Gleichaltrigen zeigen. Außerdem müssen bestimmte Bedingungen für die Studiendurchführung erfüllt sein (z.B. ausreichende Kenntnisse der deutschen Sprache bei Kindern und Eltern). Im Gesprächstermin klären wir gemeinsam ab, ob es sinnvoll ist, dass Sie und Ihr Kind an der Studie teilnehmen.

4. Wer ist der Auftraggeber der Studie und wie viele Proband/innen werden an der Studie teilnehmen?

Auftraggeber der Studie ist das Ausbildungsinstitut für Kinder- und Jugendlichenpsychotherapie der Uniklinik Köln. An der Studie nehmen insgesamt 60 Kinder zwischen 6 und 12 Jahren mit aggressivem Verhalten gegenüber Gleichaltrigen teil.

5. Wie lange dauert die Teilnahme an der Studie?

Die Therapie innerhalb der Studie umfasst etwa 16 bis 24 Patientenkontakte, zuzüglich Bezugspersonentermine d.h. im Regelfall beträgt die Gesamtdauer der Teilnahme mindestens 24 Wochen. Die Sitzungen finden einmal wöchentlich statt und dauern je 50 Minuten. Neben der Behandlung der Kinder werden auch Sie als Eltern in die Behandlung mit einbezogen. Weiterhin soll eine Lehrkraft um Teilnahme gebeten werden, um einen umfassenden Eindruck auch im Feld der Schule zu erhalten. Um untersuchen zu können, wie lang mögliche Veränderungen bei Ihnen und Ihrem Kind anhalten und Ihnen bei Bedarf die Möglichkeit einer weiterführenden Behandlung zu bieten, werden nach ca. einem Jahr weitere Nachuntersuchungen mit Ihnen und Ihrem Kind stattfinden.

6. Welches Risiko bzw. welcher Nutzen ist mit der Studienteilnahme verbunden?

Wir können nicht mit Sicherheit sagen, ob Ihr Kind und Sie von der Studienteilnahme profitieren werden. Allen durch uns angebotenen Hilfen, die in dieser Studie zum Einsatz kommen, wird durch Voruntersuchungen eine gute Wirksamkeit bescheinigt.

Die häufige, exzessive und missbräuchliche Nutzung von sogenannten „Smartdevices" (Smartphone, Tablet) kann für Kinder möglicherweise Risiken beinhalten. Während der Teilnahme an unserer Studie wird Ihr Kind jedoch die Smartphone App im Rahmen weniger Wochen, täglich nur für einen kurzen Zeitraum nutzen. Alle weiteren Funktionen des Gerätes sind zudem deaktiviert, so dass der Anreiz für eine längere Nutzung sehr gering ist. Für einen vergleichsweise kurzen Nutzungszeitraum sind uns keine Risiken bekannt.

Vor der selbständigen Nutzung im Alltag wird mit Ihrem Kind genau besprochen, wie die Therapie App anzuwenden ist, welche Art von Daten dadurch erfasst werden und welche Folgen

sich daraus für den Umgang mit dem Smartphone ergeben. Zum Schutz der Daten vor unautorisierten Personen wird das Handy durch ein Passwort geschützt.

Die Studienleitung wurde von der Ethikkommission der Medizinischen Fakultät der Universität zu Köln beraten und die Studiendurchführung durch die Ethikkommission am 12.10.2018 zustimmend bewertet.

7. Welche anderen Behandlungsmöglichkeiten gibt es außerhalb der Studie?

Sie und Ihr Kind müssen nicht an dieser Studie teilnehmen, wenn Sie oder Ihr Kind das nicht wollen. Falls Sie sich gegen eine Studienteilnahme entscheiden, werden wir Sie zu weiteren Unterstützungsangeboten beraten. Bei Kindern mit aggressivem Verhalten kann z. B. eine Beratung der Familie in einer Erziehungsberatungsstelle, eine Anbindung in einer kinder- und jugendpsychiatrischen Ambulanz oder Praxis oder eine Kinder- und Jugendlichenpsychotherapie hilfreich sein.

8. Gibt es eine spezielle Versicherung?

Da weder studienbedingte Risiken für die Gesundheit bestehen, noch studienbedingte Wege anfallen, die über die übliche Regeltherapie hinausgehen, wurden für die Studie keine Probandenversicherung und keine Wegeunfallversicherung abgeschlossen. Für die Test-Smartphones wird eine separate Geräteversicherung abgeschlossen.

9. Entstehen für mich Kosten durch die Teilnahme an der Studie?

Es entstehen die üblichen Behandlungskosten im Rahmen einer therapeutischen Intervention. Damit diese von den Krankenkassen übernommen werden, muss ein Antrag an die Kassen gestellt und von dieser bewilligt werden.

10. Ist die Studienteilnahme freiwillig?

Die Teilnahme an dieser Studie ist freiwillig. Sie ist nur möglich, wenn sowohl alle Sorgeberechtigten als auch das Kind ihre Einwilligung hierzu geben. Wenn Sie sich zur Teilnahme entscheiden und die Einwilligungserklärung unterschreiben, können Sie und Ihr Kind die Studie jederzeit, ohne Angabe von Gründen abbrechen.

Selbst wenn Sie Ihr Einverständnis geben, wird die Entscheidung Ihres Kindes respektiert, nicht an der Studie teilzunehmen bzw. die Teilnahme an der Studie vorzeitig zu beenden.

Die Projektmitarbeiter/innen werden Sie über alle neuen Erkenntnisse, Änderungen und wichtigen Informationen, die in Bezug auf diese Studie bekannt werden und die für Ihr Kind von wesentlicher Bedeutung sein könnten, umgehend informieren. Auf dieser Basis können Sie dann die Entscheidung zur weiteren Teilnahme an dieser Studie neu überdenken.

11. Was geschieht mit Ihren Daten?

Während der Studie werden Informationen von Ihnen und Ihrem Kind erhoben, niedergeschrieben und elektronisch gespeichert. Hierbei handelt es sich um personenbezogene Daten besonderer Art (über die Gesundheit, ethnische Zugehörigkeit, Geschlecht, Alter etc.). Die für die Studie wichtigen Daten werden zusätzlich in pseudonymisierter Form gespeichert, ausgewertet und gegebenenfalls weitergegeben. Pseudonymisiert bedeutet, dass keine Angaben von Namen oder Initialen verwendet werden, sondern nur ein Nummern- und/oder

Buchstabencode. Videodateien, die während der Nutzung der Therapie-App aufgezeichnet werden sind nicht im gleichen Maße unkenntlich machbar. Diese sind nur von Studienmitarbeiter/innen einsehbar und werden separat und verschlüsselt gespeichert.

Die Daten sind gegen unbefugten Zugriff gesichert. Eine Entschlüsselung erfolgt nur unter den vom Gesetz vorgeschriebenen Voraussetzungen. Die Speicherung der erhobenen Daten erfolgt in Verantwortung von Prof. Dr. Manfred Döpfner und PD Dr. Anja Görtz-Dorten, Klinik und Poliklinik für Psychiatrie, Psychosomatik und Psychotherapie des Kindes- und Jugendalters der Uniklinik Köln, Pohligstraße 9, 50969 Köln.

Die von Ihnen erhobenen Daten werden im Rahmen unserer Studie bis zu einem Widerruf rechtmäßig verarbeitet (Art. 13 Abs. 2 lit. C DSGVO). Sie haben jederzeit unentgeltlich das Recht, Einblick in Ihre Daten zu nehmen, die während der Studie erhoben werden. Sollten Sie dabei Fehler in Ihren Daten feststellen, so haben Sie das Recht, diese durch Studienmitarbeiter/innen korrigieren oder löschen zu lassen.

Die Daten, die auf der App erhoben werden, können jederzeit eigenmächtig gelöscht werden. Die Daten befinden sich lediglich auf dem Gerät, es findet keine Synchronisation mit Servern oder ähnliche Übertragung statt.

Weitere Einzelheiten, insbesondere zur Möglichkeit eines Widerrufs, entnehmen Sie bitte der Einwilligungserklärung im Anschluss an diese Studieninformation.

Die Mitwirkung an der Datenerhebung ist freiwillig. Sollten Sie Bedenken haben oder nicht zustimmen können wir aufgrund des Studiendesigns eine Studienteilnahme nicht ermöglichen. Wir werden Sie in diesem Falle gerne zu weiteren Unterstützungsmöglichkeiten beraten (siehe auch Punkt 7).

12. An wen wende ich mich bei weiteren Fragen?

Für weitere Fragen im Zusammenhang mit dieser Studie stehen Ihnen die Projektmitarbeiter/innen gerne zur Verfügung. Auch Fragen, welche die Rechte Ihres Kindes als Teilnehmer/in an dieser Studie oder die Aufbewahrung bzw. Vernichtung von persönlichen Daten betreffen, werden wir Ihnen gerne beantworten. Wenn Sie, Ihr Kind oder Ihre Angehörigen Fragen oder Bedenken zu dieser Studie, zu möglichen Risiken und unerwünschten Nebenwirkungen der eingesetzten Formen der Hilfe oder zu Ihren Rechten haben oder wenn Sie zusätzliche Informationen wünschen oder studienbezogene Schädigungen melden möchten, können Sie und Ihr Kind jederzeit die Projektmitarbeiter/innen kontaktieren.

**Kontakt Studienleitung**
AUTHARK-Team<br><br>Ausbildungsinstitut für Kinder- und Jugendlichenpsychotherapie<br>an der Uniklinik Köln<br>Pohligstr. 9<br>50969 Köln<br><br>Tel. Fr. Fessel: +49 221 478 76809<br>Tel. Fr. Frank: +49 221 478 30952<br>Tel. Fr. Hofmann: +49 221 478 30953<br>E-Mail: authark@uk-koeln.de

Ihr Team von AUTHARK

Forschungsprojekt AUTHARK

- Einwilligungserklärung für Eltern –

Hilfen für Kinder mit aggressivem Verhalten und affektiver Dysregulation

Einen Flyer mit Informationen zu unserer Studie haben Sie von uns erhalten und wir haben mit Ihnen persönlich und ausführlich über den Studienablauf gesprochen. Mit Ihrer Unterschrift bestätigen Sie uns die folgenden Aussagen:

- Ich habe die Probandeninformation (Flyer) erhalten und gelesen und Ziel, Ablauf und Durchführung der Studie verstanden. Ich wurde über Wesen, Bedeutung, Tragweite und Risiken der geplanten Studienteilnahme informiert. Mir wurde ausreichend Gelegenheit gegeben, alle offenen Fragen zu klären. Ich habe jederzeit das Recht, weitere Informationen zur Studie zu erhalten.
- Ich bin bereit, an der Studie teilzunehmen und zu vier Zeitpunkten Fragebogen auszufüllen und jeweils an den Interviewterminen teilzunehmen. Mein Kind füllt ebenfalls Fragebogen aus und nimmt an Interviews teil.
- Ich bin damit einverstanden, dass ich über eine Zufallszuweisung (Randomisierung) einer Interventionsgruppe (THAV/ScouT oder THAV/ ScouT +AUTHARK) zugeordnet werde.
- Wenn ich in die Gruppe THAV/ ScouT+ AUTHARK gelost werde, bin ich damit einverstanden, dass mein Kind ein extra für die Studie aufgesetztes Test-Smartphone nutzt und werde es daran erinnern, dieses pfleglich zu behandeln.
- Wenn ich in die Gruppe THAV/ ScouT +AUTHARK gelost werde, weise ich mein Kind daraufhin, dass es bei den Aufnahmen für das Videotagebuch nur sich selbst und keine weiteren Personen filmt.
- Ich habe jederzeit das Recht, ohne Angabe von Gründen von der Teilnahme an der Studie zurückzutreten, ohne dass dadurch Nachteile für mich oder mein Kind entstehen. In diesem Fall werden die bis zu diesem Zeitpunkt erhobenen Daten gelöscht oder vollständig anonymisiert, es sei denn, ich stimme/wir stimmen nach erfolgtem Widerruf der weiteren Verwendung der bislang erhobenen Daten ausdrücklich zu.
- Ich wurde darüber informiert, dass die Interviews im Rahmen der Messzeitpunkte auf Video aufgezeichnet werden.
- Zu Verarbeitung und zum Schutz unserer Daten wurden mir die in der hier nachfolgend abgedruckten Datenschutzerklärung stehenden Zusagen gemacht.

Datenschutz:

Mir/Uns ist bekannt, dass bei dieser Studie personenbezogene Daten über mich/uns und mein/unser Kind erhoben, gespeichert und ausgewertet werden sollen. Dabei handelt es sich insbesondere um medizinische Befunde des Kindes.

Die Verwendung der Angaben über die Gesundheit des Kindes erfolgt nach gesetzlichen Bestimmungen und setzt vor der Teilnahme an der Studie folgende freiwillig abgegebene Einwilligungserklärung voraus, das heißt, ohne die nachfolgende Einwilligung kann das Kind nicht an der Studie teilnehmen.

Unsere persönlichen Daten werden in Übereinstimmung mit dem geltenden Datenschutzgesetz vertraulich behandelt. Sie werden in pseudonymisierter Form gespeichert. Das bedeutet, dass Identifikationsmerkmale (z.B. Name, Anschrift) durch eine Codenummer ersetzt werden, sodass eine Zuordnung der Fragebogendaten zu einer bestimmten Person nur über weitere Hilfsmittel (Referenzliste) möglich ist. Diese Verbindung wird lediglich für die Koordination der Erhebung benötigt und anschließend vernichtet. Die wissenschaftliche Auswertung der Daten durch den Sponsor (Universität zu Köln) oder einer von diesem beauftragte Stelle erfolgt so, dass kein Rückschluss auf mich oder mein Kind möglich ist. Ein Rückschluss ist nur dann theoretisch möglich, wenn die Person, die die Daten auswertet, auch über die Referenzliste zum Entschlüsseln der Pseudonymisierung verfügt. Mir ist bewusst, dass die Aufnahmen, die mein Kind im Rahmen der App-Nutzung bei der Aufzeichnung des Video-Tagebuchs tätigt, nicht in der gleichen Form wie die übrigen Daten unkenntlich gemacht werden können. Diese Daten werden jedoch nur von autorisierten Mitarbeitern der Studie zur wissenschaftlichen Auswertung eingesehen und unter Einhaltung besonderer Schutzmaßnahmen verschlüsselt separat gespeichert. Von den Ergebnissen der Auswertung kann kein Rückschluss mehr auf mein Kind oder mich gezogen werden. Im Falle der Veröffentlichung von Studienergebnissen bleibt die Vertraulichkeit unserer persönlichen Daten damit gewährleistet.

Die im Rahmen dieser Studie erhobenen personenbezogenen Daten, insbesondere Angaben über die Gesundheit des Kindes, werden von einem elektronischen Datensystem erfasst und ausgewertet. Nach Ende der Studie werden alle Daten nach den derzeit gültigen Richtlinien entsprechend gespeichert, archiviert und für eine weitere Verwendung gesperrt.

Ich habe das Recht, Einblick in die zu mir und meinem Kind im Rahmen der Studie erfassten personenbezogenen Daten zu nehmen sowie eine unentgeltliche Kopie zu erhalten. Sollte ich dabei Fehler feststellen, habe ich das Recht, diese korrigieren oder löschen zu lassen. Die gespeicherten Daten werden bis zum Widerruf rechtmäßig verarbeitet (Art. 13 Abs. 2 lit. C DSGVO). Bereits gespeicherte, insbesondere personenbezogene Daten, die nicht sofort gelöscht werden können, werden gegen eine weitere Nutzung gesperrt (Art. 13 Abs. 2 lit. B DSGVO; Art. 18 Abs. 1 DSGVO). Unsere Daten werden über einen Zeitraum von mindestens 10 Jahren in einem sicheren System gespeichert und im Anschluss gelöscht. Die Beachtung des Bundesdatenschutzgesetzes ist in vollem Umfang sichergestellt.

Ich habe das Recht auf Widerruf hinsichtlich der Datenverarbeitung. In diesem Fall werden die bis zu diesem Zeitpunkt erhobenen Daten gelöscht oder vollständig anonymisiert und für die weitere Bearbeitung gesperrt (Art. 13 Abs. 2 lit. B DSGVO; Art. 18 Abs. 1 DSGVO), es sei denn, ich stimme/wir stimmen nach erfolgtem Widerruf der weiteren Verwendung der bislang erhobenen Daten ausdrücklich zu. Ich bin/wir sind bereits darüber aufgeklärt worden, dass das Kind die Teilnahme an der Studie jederzeit beenden kann.

Ich erkläre mich damit einverstanden, dass autorisierte und zur Verschwiegenheit verpflichtete Beauftragte des Sponsors zum Zwecke der Qualitätssicherung sowie Beauftrage der Ethikkommission zum Zwecke des Monitorings in die beim Studienleiter vorhandenen personenbezogenen Daten meines Kindes Einsicht nehmen, soweit dies für die Überprüfung der ordnungsgemäßen Durchführung der Studie notwendig ist. Für diese Maßnahme entbinde ich die im Rahmen der Studie für mich bzw. mein Kind zuständigen Studienmitarbeiter von der ärztlichen Schweigepflicht.

Die Speicherung und Auswertung der erhobenen Daten erfolgt in Verantwortung von Univ.-Prof. Dr. Manfred Döpfner Klinik und Poliklinik für Psychiatrie, Psychosomatik und Psychotherapie des Kindes- und Jugendalters der Uniklinik Köln, Pohligstraße 9, 50969 Köln

Der zuständige Datenschutzbeauftrage für die Universität zu Köln ist Herr Alexander May, LL.M., Albertus Magnus Platz, 50923 Köln

Mir ist bekannt, dass ich berechtigt bin, mich bei einer Datenschutz-Aufsichtsbehörde zu beschweren. Ich kann mich in diesem Fall bei der Landesbeauftragen für Datenschutz und Informationsfreiheit Nordrhein-Westfalen (Landesbeauftragte für den Datenschutz und Informationsfreiheit, Postfach 20 04 44, 40102 Düsseldorf, Tel.: 0211/38424-0, Mail: poststelle@ldi.nrw.de) beschweren.

Haben Sie noch Fragen? Wenden Sie sich an uns - wir beantworten alle Ihre Fragen sehr gern!

- Einwilligungserklärung für Eltern (AUTHARK)-

Ich habe die obigen Informationen gelesen. ☐ ja ☐ nein

Ich habe die obige Datenschutzerklärung gelesen und bin mit dem Sachverhalt einverstanden

☐ ja ☐ nein

- Ich habe verstanden, warum die Studie gemacht wird und auch, was gemacht wird. Ich kann jederzeit weitere Informationen zur Studie bekommen.
- Ich bin damit einverstanden, dass ich über Zufallsverteilung (Randomisierung) einer von zwei Gruppen (THAV/ ScouT oder THAV/ ScouT +AUTHARK) zugeordnet werde.
- Wenn mein Kind in die Gruppe THAV/ ScouT +AUTHARK gelost werde, bin ich damit einverstanden, dass mein Kind für einen gewissen Zeitraum ein Test-Smartphone nutzt, welches extra für die Studie präpariert wurde. Darüber hinaus bin ich dazu bereit einen separaten Leihvertrag zu unterzeichnen.
- Ich wurde darauf hingewiesen, dass bei den Aufzeichnungen der Video-Tagebuchfunktion in der Smartphone App, nur mein Kind und keine weiteren Personen gefilmt werden sollen. Auch mit meinem Kind wurde dies besprochen.
- Ich möchte an der Studie teilnehmen und weiß, dass dies freiwillig ist. Ich kann auch später zu jeder Zeit sagen, wenn ich nicht mehr teilnehmen möchte. Mir oder meinem Kind werden dadurch keine Nachteile entstehen.
- Ich bin damit einverstanden, dass mein Kind an der Studie teilnimmt.
- Meine Daten werden verschlüsselt unter einem Code gespeichert und untersucht.
- Ich bin damit einverstanden, dass die in der App aufgenommenen Video- und Textdateien gespeichert und ausgewertet werden (bitte zutreffendes ankreuzen).

☐ ja — Ich habe verstanden, dass es nicht möglich ist, Videodaten so zu verändern, dass nicht mehr erkennbar ist, welche Person auf dem Video zu sehen ist. Ich weiß, dass die Videodateien nur von autorisierten Mitarbeitern der Studie gesehen werden dürfen.

☐ nein

- Mein Kind hat der Teilnahme an der Studie nach einer umfassenden Aufklärung ebenfalls zugestimmt und eine entsprechende Einwilligungserklärung unterschrieben

☐ ja ☐ nein

____

Name des Kindes in Druckbuchstaben

☐ Ich entbinde/Wir entbinden das Studienpersonal von der Schweigepflicht gegenüber dem/r Lehrer/in ________________________________ (Name der Lehrkraft, die in die Behandlung einbezogen werden soll) und erkläre mich/erklären uns damit einverstanden, dass der/die Lehrer/in gegenüber dem Studienpersonal Informationen bezüglich des Verhaltens des Kindes weitergeben darf.

Für die Teilnahme des Kindes an der Studie ist die Zustimmung beider Sorgeberechtigter erforderlich. Falls Sie das alleinige Sorgerecht besitzen, kreuzen Sie bitte hier an:

o Ja, ich habe die Alleinsorge für das genannte Kind.

Ort, Datum

Name & Unterschrift **erste/r Sorgeberechtigte/r**

Ort, Datum

Name & Unterschrift **zweite/r Sorgeberechtigte/r**
(ggf. durchstreichen)

Ich habe das Aufklärungsgespräch geführt und die Einwilligung der Erziehungsberechtigten eingeholt.

Name des/r Studienmitarbeiter/in in Druckbuchstaben

Datum

Unterschrift des/der aufklärenden Studienmitarbeiters/in
